# Antigenic remodeling of the SARS-CoV-2 Spike N-terminal domain links sequence variability to structural reorganization of a neutralizing antibody supersite

**DOI:** 10.3389/fcimb.2026.1924817

**Published:** 2026-09-14

**Authors:** Juliana Santa Ardisson, Brena Ramos Athaydes, Mariane Vedovatti Monfardini Sagrillo, Raphaella Luisa Fernandes de Almeida, Greiciane Gaburro Paneto, Hebert de Paula, Gabriela Ferreira Nunes, Liliana Cruz Spano, Sandra Ventorin von Zeidler, Teodiano Freire Bastos-Filho

**Affiliations:** 1 Postgraduate Program in Biotechnology, Health Sciences Center, Federal University of Espírito Santo, Vitória, Espírito Santo, Brazil; 2 Department of Pharmacy and Nutrition, Center of Exact, Natural and Health Sciences, Federal University of Espírito Santo, Alegre, Espírito Santo, Brazil; 3 Postgraduate Program in Infectious Diseases, Health Sciences Center, Federal University of Espírito Santo, Vitória, Espírito Santo, Brazil; 4 Department of Pathology, Health Sciences Center, Federal University of Espírito Santo, Vitória, Espírito Santo, Brazil

**Keywords:** antibody–antigen interface, antigenic remodeling, genomic surveillance, molecular dynamics, neutralizing antibody supersite, N-terminal domain, SARS-CoV-2

## Abstract

**Introduction:**

The N-terminal domain (NTD) of the Severe Acute Respiratory Syndrome Coronavirus 2 (SARS-CoV-2) Spike harbors a neutralizing antibody supersite and is a recurrent site of antigenic change. However, how sequence variability across pre- and post-vaccination immune landscapes reshapes antibody recognition remains incompletely characterized.

**Methods:**

Clinical-genomic surveillance was combined with structural modeling and molecular dynamics to identify antigenically relevant NTD changes and probe their structural impact on the neutralizing antibody interface. SARS-CoV-2 profiles were compared across two periods reflecting contrasting immune contexts: a pre-vaccination period dominated by B.1.1.33 and a post-vaccination period dominated by the Omicron sublineage BQ.1.1. A local cohort (n=126) supported descriptive characterization. An expanded cohort integrating local and GISAID genomes with harmonized clinical metadata (n=534; 401 pre-vaccination, 133 post-vaccination) was used to prioritize candidate loci by their association with clinical severity.

**Results:**

In adjusted models, age remained independently associated with severity in both periods, whereas sex did not. In the pre-vaccination cohort, Spike S in the NTD was associated with greater severity, without phylogenetic segregation by outcome. This association is exploratory and was used to prioritize an antigenically located NTD change for structural analysis. Q23K was regionally concentrated in Espírito Santo (57.4%) relative to other Brazilian states (2.7%) and international genomes (1.4%). In the post-vaccination cohort, an overlapping deletion event (ORF9b -29/N -33) was associated with non-severe disease, but should be interpreted cautiously given metadata heterogeneity, cohort structure, and the nature of the locus. However, in this period clinical outcome and sequencing source were partially confounded. A source-stratified sensitivity analysis restricted to GISAID-only samples showed the association persisted, but could not rule out a source- or pipeline-dependent indel-calling artifact, so the finding is reported as exploratory. Although Q23K itself did not persist, adjacent NTD changes (T19I/A27S/D24 -26) became recurrent post-vaccination, indicating remodeling of the same antigenic neighborhood rather than persistence of a single mutation. Structural modeling and molecular dynamics of the NTD-antibody 1 -87 complex supported reorganization of the modeled antibody-antigen interface, with redistribution of supersite contacts rather than loss of structural compatibility.

**Discussion:**

These findings link NTD variability across immune contexts to structural reorganization of the modeled antibody-antigen interface and support integrating genomic surveillance with structural analysis to prioritize antigenically relevant changes for functional testing.

## 1 Introduction

Viral evolution is a continuous process that challenges outbreak prediction and control, as adaptive changes can alter transmissibility, antigenicity, and disease severity over short time scales (Jacobs et al., 2023). In RNA viruses, this dynamic is favored by high replication rates, large population sizes, and the combined effects of genetic drift and selection. In coronaviruses, although proofreading mechanisms increase replication fidelity compared with other RNA viruses, sustained circulation under distinct selective pressures maintains substantial diversification, including recombination in specific settings (Denison et al., 2011; Focosi and Maggi, 2022; Gribble et al., 2021).

The COVID-19 pandemic exemplified this process on a global scale, with successive waves associated with the emergence and replacement of variants of concern (VOCs), affecting transmissibility, immune escape, and the performance of antibody-based interventions (Jacobs et al., 2023). These changes were driven primarily by mutational patterns in the Spike protein, the main determinant of viral entry and a principal target of neutralizing antibodies (Wrapp et al., 2020). Although the receptor-binding domain (RBD) has received most attention, the N-terminal domain (NTD) is also a major antigenic region. The NTD harbors a supersite targeted by potent neutralizing antibodies and is a hotspot for recurrent deletions and substitutions, which recur across variants and are frequently associated with antibody escape (Cerutti et al., 2021; McCallum et al., 2021). Because these changes recur at the same antigenic site, the NTD supersite is informative for monitoring how Spike variability affects antibody recognition. Studies integrating NTD mutational signatures from clinical samples with structural analysis of the antibody interface, across changing epidemiological and immunological contexts, remain limited.

SARS-CoV-2 evolution is shaped primarily by selection for efficient transmission and immune escape, whereas changes in disease severity are not considered a direct target of viral selection (Markov et al., 2023; Carabelli et al., 2023). As population immunity increased through vaccination and prior infection, the selective landscape also changed, favoring viral adaptations that improve fitness in an increasingly immune host population (Carabelli et al., 2023; Harvey et al., 2021). Thus, differences in average disease severity across pandemic periods likely reflect the combined effects of population immunity and the intrinsic properties of circulating lineages, rather than a simple evolutionary shift in viral virulence (Carabelli et al., 2023). Within this framework, associations between individual mutations and disease severity are not interpreted as evidence of increased viral virulence. Instead, severity strata were used to prioritize candidate changes located in antigenically relevant regions for structural analysis, with observed associations interpreted as context-dependent epidemiological markers rather than causal determinants of clinical outcome.

In addition to changes in transmissibility and immune escape, the pandemic was marked by broad clinical heterogeneity, ranging from asymptomatic infection to severe disease and death. Because disease severity results from the interaction between host factors and viral characteristics, integrating clinical and genomic data is essential to investigate whether mutational signatures are associated with clinical phenotypes across different epidemiological phases (Fourati et al., 2022). In Brazil, the start of mass vaccination in January 2021 defined a relevant transition between an initial period of greater population susceptibility and a later phase characterized by widespread vaccination and prior viral exposure (Li et al., 2024), providing a useful context to examine how selective pressures and mutational profiles of SARS-CoV-2 changed over time (Fourati et al., 2022; Jacobs et al., 2023).

Structural modeling and molecular dynamics can test the conformational plausibility of mutation effects on antibody-antigen interfaces, complementing sequence-level surveillance. The present study combined clinical, genomic, and structural data to examine how NTD sequence variability relates to antibody recognition at the NTD supersite across two periods with contrasting immune contexts: pre-vaccination circulation of B.1.1.33 and post-vaccination circulation of the Omicron sublineage BQ.1.1. The local cohort was used to describe each period. The expanded cohort, with harmonized clinical metadata, was used to prioritize candidate NTD changes according to their association with clinical severity. These changes were then evaluated at the interface with the neutralizing antibody *1–87* by structural modeling and molecular dynamics, as a test of conformational plausibility for altered antigenic recognition. This combination of severity-prioritized surveillance and structural analysis links circulating NTD variability to potential changes in neutralizing antibody recognition across distinct immune contexts.

## 2 Materials and methods

Detailed technical and operational procedures are provided in the **Supplementary Methods** (Sections S1-S7), **Supplementary Tables 1**–**S7**, and **Supplementary Figures 1**–**S7**.

### 2.1 Study design, time periods, and clinical definitions

Naso/oropharyngeal swab samples positive for SARS-CoV-2 by RT-qPCR were analyzed from Espírito Santo state, southeastern Brazil, across two time windows: a pre-vaccination period (July 9, 2020 to March 29, 2021) and a post-vaccination period (November 14, 2022 to December 28, 2022). The post-vaccination period corresponded to a phase after vaccine rollout in Brazil, during which Omicron sublineages were circulating (Li et al., 2024; Santos et al., 2022).

Two analytical datasets were defined. The local cohort comprised locally sequenced samples and was used exclusively for descriptive clinical-genomic characterization. The expanded cohort, integrating local genomes and public GISAID genomes with harmonized clinical metadata, selected according to predefined eligibility criteria (**Supplementary Methods S4**), was used for inferential analyses of severity-associated mutational signals. Public genomes and associated metadata included in the expanded cohorts were organized into persistent GISAID datasets identified as EPI_SET_260306xf (pre-vaccination expanded cohort; DOI: 10.55876/gis8.260306xf) and EPI_SET_260306wr (post-vaccination expanded cohort; DOI: 10.55876/gis8.260306wr). The dataset used for phylogenetic reconstruction was archived separately as EPI_SET_260125qx (DOI: 10.55876/gis8.260125qx).

Clinical severity was operationally classified into two subgroups. In the local cohort, severe cases were defined by ICU admission, whereas mild cases were defined as asymptomatic or symptomatic outpatients, not requiring hospitalization. In the expanded cohort, severity categories were harmonized from patient-status metadata into a binary variable (mild/non-severe vs severe/hospital-based) using predefined curation rules (**Supplementary Table 2**). In the local post-vaccination cohort only mild cases were available, and severe post-vaccination cases were incorporated only at the expanded-cohort stage using public GISAID genomes with clinical metadata. In the resulting expanded cohort, mild cases originated from both local sequencing (n=30) and GISAID (n=39), whereas all severe cases (n=64) were obtained from GISAID; outcome and sequence source are therefore partially confounded in the post-vaccination period, and severity associations in this period are interpreted with this limitation in mind (see Discussion). In the pre-vaccination expanded cohort, by contrast, both severity strata included locally sequenced and GISAID-derived genomes (severe: 26 local, 222 GISAID; mild: 6 local, 147 GISAID), so clinical outcome and sequencing source were not confounded in that period.

### 2.2 RNA extraction, RT-qPCR, whole-genome sequencing, and quality control

RNA extraction, RT-qPCR screening, tiled amplicon generation using the Midnight protocol, Oxford Nanopore sequencing, and primary bioinformatic processing were performed largely as previously described (Santa Ardisson et al., 2025). Detailed laboratory and bioinformatic procedures, including supplementary primers used for Omicron samples, are provided in **Supplementary Methods S2**, **S3** and **Supplementary Table 1**.

### 2.3 Dataset construction

Initial clinical-epidemiologic analyses were performed in the local cohort, comprising locally sequenced samples with ≥90% whole-genome coverage, regardless of lineage. Based on lineage distribution in the local cohort, the predominant lineages in each period were selected for the expanded analyses: B.1.1.33 in the pre-vaccination period and BQ.1.1 in the post-vaccination period.

The expanded cohort integrated local genomes and public GISAID genomes and required: (i) ≥90% whole-genome coverage, (ii) ≥90% Spike coverage, and (iii) for public genomes, availability of clinical metadata with patient status. Severity categories were harmonized before analysis (**Supplementary Methods S4**; **Supplementary Table 2**). The final expanded dataset included 401 genomes in the pre-vaccination period (248 severe; 153 mild) and 133 genomes in the post-vaccination period (64 severe; 69 mild). Sample selection and dataset composition are summarized in **Supplementary Figure 1**. For age- and sex-adjusted models, only records with both covariates available were included, resulting in an effective N = 388 (pre-vaccination: 237 severe; 151 mild) and N = 131 (post-vaccination: 63 severe; 68 mild).

### 2.4 Phylogenetic analysis of Q23K

To investigate the phylogenetic context of Q23K during circulation of lineage B.1.1.33, a dataset was built including all local Q23K-positive sequences (30 sequences) and public GISAID sequences selected by similarity within the same time interval (156 sequences). The Wuhan-Hu-1 reference genome (NC_045512.2) (Wu et al., 2020) was included for reference and tree rooting. Sequences were aligned with MAFFT (Katoh and Standley, 2013), and maximum-likelihood phylogenetic inference was performed in IQ-TREE v1.6.12 (Nguyen et al., 2015) with automatic model selection by ModelFinder (GTR+F+I, BIC) (Kalyaanamoorthy et al., 2017), and branch support estimated using Ultrafast Bootstrap (1000 replicates). The consensus tree was inspected and annotated in iTOL. Detailed dataset selection parameters are provided in **Supplementary Methods S5**.

Independent of the phylogenetic dataset used for contextualizing Q23K-positive genomes (Section 2.4), the prevalence of S:Q23K among B.1.1.33 genomes was estimated separately for Espírito Santo, other Brazilian states, and internationally using a systematic GISAID query (**Supplementary Methods S5.2**).

### 2.5 Structural modeling and molecular dynamics of the NTD-*1–87* complex

The monoclonal antibody *1-87*, originally isolated from a convalescent donor and described by Liu et al. (2020), was selected as a structural probe because it recognizes the NTD antigenic supersite and has an experimentally resolved Spike-bound structure (PDB 7L2D), enabling structural modeling and molecular dynamics (MD) with a defined conformational reference. The *1–87* antibody was used as a biologically relevant structural probe and was not intended to represent the full diversity of the anti-NTD humoral response (Cerutti et al., 2021).

The reference structure was obtained from the RCSB PDB (7L2D). The modeled Spike fragment corresponded to the NTD and adjacent region (residues S:14-320, Wuhan-Hu-1 numbering). The NTD chain was modeled using AlphaFold3 (Abramson et al., 2024), and two variants were generated from the wild-type (WT) model: Q23K (pre-vaccination representative) and the T19I/A27S/Δ24–26 motif, hereafter referred to as LPP (post-vaccination representative). The NTD-*1–87* complexes used as starting structures for molecular dynamics were subsequently assembled by least-squares superposition of the predicted NTD core onto the NTD present in the experimentally resolved 7L2D complex, while retaining the experimental coordinates and orientation of the *1–87* Fab. This template-guided strategy was selected to preserve a common experimentally observed antibody-binding pose and identical initial Fab geometry across WT, Q23K, and LPP. It thereby reduced variation caused by independently predicted antibody-docking orientations and allowed the comparative analysis to focus on the effects of NTD sequence variation. The assembled complexes were subsequently subjected to energy minimization and molecular-dynamics equilibration before production simulations. Molecular dynamics simulations were performed in GROMACS (Abraham et al., 2015, 2024), using the CHARMM36 force field (Best et al., 2012). For each condition (WT, Q23K, and LPP), three independent 200-ns replicas were run, totaling 600 ns per condition.

Trajectory analyses included root-mean-square deviation (RMSD), root-mean-square fluctuation (RMSF), solvent-accessible surface area (SASA), and radius of gyration (Rg), as well as predicted interfacial interactions using PLIP (Salentin et al., 2015). For comparative frame-wise affinity estimates, frames were processed with PDBFixer (OpenMM, 2026) and submitted to PRODIGY (Xue et al., 2016) to estimate binding free energy (ΔG) and dissociation constant (Kd). Buried surface area (BSA) was calculated using FreeSASA (Mitternacht, 2016). Complete structural modeling, simulation, and post-processing details are described in **Supplementary Methods S6**.

Per-atom predicted Local Distance Difference Test scores (pLDDT) were extracted from the AlphaFold 3.json output and mapped to the corresponding atoms and residues in the model CIF file. Residue-level confidence was represented by the pLDDT value assigned to the Cα atom of each amino-acid residue. Mean, standard deviation, median, and range of residue-level pLDDT values were calculated for the complete modeled region and separately for the N3 (residues 141–156) and N5 (residues 246–260) loops.

### 2.6 Statistical analysis

The local cohort was used exclusively for descriptive clinical-genomic characterization, without inferential testing or regression modeling. Accordingly, all inferential statistical analyses reported in this study refer to the expanded cohort.

Continuous clinical variables were summarized as median and interquartile range (IQR), categorical variables as counts and percentages, MD-derived parameters were summarized primarily as median and IQR, whereas mean ± SD values were additionally reported for descriptive comparison with the time-resolved trajectory plots. Normality of continuous variables was assessed using the Shapiro-Wilk test. Because at least one comparison group showed evidence of non-normality, comparisons between independent groups were performed using the Mann-Whitney U test for continuous variables. Categorical variables were compared using the Chi-square test or Fisher’s exact test, as appropriate. A two-sided significance threshold of α=0.05 was adopted.

Associations between mutations and clinical severity were evaluated separately within each period-specific expanded cohort using binary logistic regression, with harmonized severity as the outcome (0=mild; 1=severe). To reduce multiple testing and instability associated with rare or near-fixed variants, candidate mutations were first screened within each cohort using the subset with known age, sex, and severity. Only mutations with prevalence > 5% were considered eligible (**Supplementary Methods S7**; **Supplementary Table 3**). These mutations were first explored in unadjusted analyses as part of an exploratory screening step aimed at prioritizing candidate loci for adjusted modeling. This screening was used to reduce redundancy and instability among multiple mutation candidates within each cohort, rather than as a strict hypothesis-testing gatekeeper. Mutations showing sufficient variation and an initial signal in this exploratory stage were then prioritized for multivariable logistic regression.

In adjusted models, mutation status (0=absent; 1=present) was included as the main predictor, and age and sex were included as covariates. Age was modeled per 10-year increase. Adjusted analyses were restricted to records with known age, sex, and severity. For each cohort, the mutation(s) prioritized during this screening step were entered into an age- and sex-adjusted logistic regression model, from which odds ratios (ORs), 95% confidence intervals (95% CIs), and p values were obtained (**Supplementary Table 3**). For variants exhibiting quasi-complete separation under standard maximum-likelihood estimation, Firth’s penalized (bias-reduced) logistic regression was used instead to obtain finite, interpretable estimates, details are provided in **Supplementary Table 3**. To formally account for multiple testing across the mutations meeting the prevalence threshold in each cohort, Benjamini-Hochberg FDR correction was applied to the unadjusted screening p-values; corrected q-values are reported in **Supplementary Table 3**.

For molecular dynamics–derived parameters (SASA, BSA, and ΔG), normality was assessed using the Shapiro-Wilk test. As WT, Q23K, and LPP were simulated as independent systems, frame-wise measurements were not paired across conditions; thus, comparisons were performed using the Kruskal-Wallis test followed by Dunn’s *post hoc* test with Benjamini-Hochberg adjustment. The WT complex was used as the reference group in GraphPad Prism (version 9.0.0.121; GraphPad Software, San Diego, CA, USA). Effect sizes were quantified using epsilon squared (ϵ²) for the omnibus test, and Cliff’s delta (δ) for the prespecified Dunn *post hoc* comparisons against WT. To reflect the full triplicate design (600 ns/condition), frame-wise distributions were pooled across the three independent 200-ns replicates per condition (n = 6,003 frames/condition per test, covering the full 200-ns trajectories). Descriptive summaries reported in **Figure 1** and **Supplementary Table 5** refer instead to the production phase (100–200 ns) and are expressed as the mean ± SD across the three replicate means (n = 3 replicates); convergence and inter-replicate reproducibility were additionally assessed via block-averaging (10 blocks of 20 ns) and cumulative-mean analysis (**Supplementary Methods S7.1**; **Supplementary Tables 5**, **S6**). Inferential analyses were performed in IBM SPSS Statistics for Windows, version 21.0 (IBM Corp., Armonk, NY, USA). Descriptive statistics and figures were generated in Python 3.10 and R 4.3.2.

**Figure 1:**
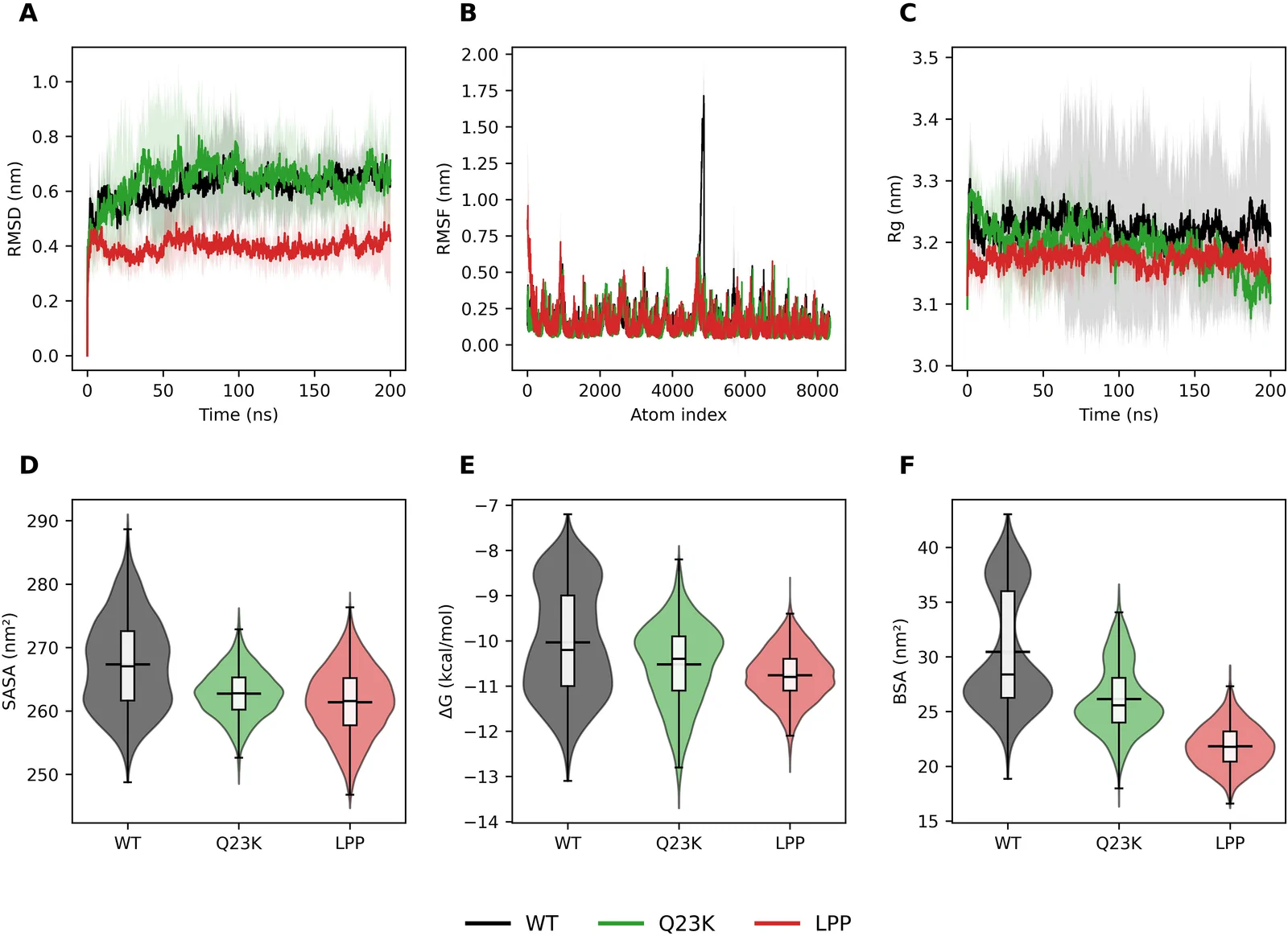
Time-resolved structural and interface metrics of the NTD-*1–87* complex in WT, Q23K, and LPP models. Time-resolved structural and interface metrics of the NTD-*1-87* complex in WT, Q23K, and LPP models, pooled across three independent 200-ns replicates per condition (600 ns/condition). Shaded bands represent the standard deviation across the three independent replicates; solid lines represent the replicate-averaged trace. **(A)** Root-mean square deviation (RMSD, nm) of the NTD after least-squares alignment; mean ± SD across the pooled replicates: WT, 0.64 ± 0.11 nm; Q23K, 0.64 ± 0.13 nm; LPP, 0.40 ± 0.07 nm. **(B)** Root-mean-square fluctuation (RMSF, nm) per atom. **(C)** Radius of gyration (Rg, nm); mean ± SD: WT, 3.22 ± 0.14 nm; Q23K, 3.17 ± 0.01 nm; LPP, 3.17 ± 0.03 nm. **(D)** Solvent-accessible surface area (SASA, nm²); mean ± SD: WT, 267.3 ± 6.9 nm²; Q23K, 262.3 ± 2.04 nm²; LPP, 261.0 ± 5.9 nm². **(E)** PRODIGY-predicted binding free energy (ΔG, kcal/mol); mean ± SD: WT, −10.0 ± 1.1 kcal/mol; Q23K, −10.6 ± 0.7 kcal/mol; LPP, −10.8 ± 0.4 kcal/mol. **(F)** Buried surface area (BSA, nm²); mean ± SD: WT, 30.5 ± 6.42 nm²; Q23K, 26.0 ± 3.3 nm²; LPP, 21.7 ± 2.0 nm². Colors indicate WT (black), Q23K (green), and LPP (red). Frame-wise distributions of SASA, ΔG, and BSA (n = 6,003 frames/condition, pooled across replicates) were compared using Kruskal-Wallis tests followed by Dunn’s *post hoc* comparisons using WT as the reference group, with Benjamini-Hochberg adjustment. Global effect sizes were quantified using epsilon squared (ϵ²), and pairwise effect sizes using Cliff’s delta (δ). All omnibus tests and WT-referenced comparisons remained significant at adjusted p < 0.0001.

### 2.7 Ethics statement

The study was conducted in accordance with the ethical standards of the Declaration of Helsinki and was approved by the Research Ethics Committee of the Federal University of Espírito Santo (UFES), Brazil, under approval no. 6.173.702.

## 3 Results

### 3.1 Clinical and epidemiological characterization of the local cohort

Clinical and demographic characteristics of the local cohort are summarized in **Table 1**. The pre-vaccination set included 53 genomes, comprising 11 mild and 42 severe cases, whereas the post-vaccination set included 73 mild cases only. The pre-vaccination cohort was older overall than the post-vaccination cohort, largely reflecting the higher proportion of severe cases. Within the pre-vaccination period, severe cases were older than mild cases. Women predominated in the post-vaccination cohort (72.6%), whereas sex distribution was more balanced in the pre-vaccination cohort. Self-reported race/color is presented in **Table 1**.

**Table 1:** Demographic, clinical, and laboratory characteristics of the local SARS-CoV-2 cohort included in genomic analyses, stratified by sampling period and clinical severity.

Variable		Pre-vaccination Cohort			Post-vaccination Cohort
		Total (n=53)	Mild (n=11)	Severe (n=42)	Total n=73
Sex, female % (n)		45.3% (24/53)	63.6% (7/11)	40.5% (17/42)	72.6% (53/73)
Sex, male % (n)		54.7% (29/53)	36.4% (4/11)	59.5% (25/42)	27.4% (20/73)
Age, years -median (IQR)		63 (40-75)	42 (29.5-60.0)	66.5 (45.5-76.0)	40 (30-51)
Self-declared race/color	White %(n)	34.0% (18/53)	81.9% (9/11)	21.4% (9/42)	38.4% (28/73)
	Brown %(n)	34.0% (18/53)	18.2% (2/11)	38.1% (16/42)	48.0% (35/73)
	Black %(n)	1.9% (1/53)	0.0%	2.4% (1/42)	12.3% (9/73)
	Not Informed %(n)	30.2% (16/53)	0.0%	38.1% (16/42)	1.4% (1/73)
Ct value (N2 gene) - median (IQR)		21.0 (19.0-23.5)	21.9 (18.0-25.1)	20.9 (19.1-23.0)	18.3 (16.7-21.2)
Hospital stay, days - median (IQR)^a^		N/A	N/A	16.5 (10.5-27.5)	N/A
Death outcome % (n)^b^		N/A	N/A	54.8% (23/42)	N/A

Data are shown as median (IQR) for continuous variables and n (%) for categorical variables. The pre-vaccination cohort (n = 53) was stratified into mild (n = 11) and severe (n = 42) cases, whereas the post-vaccination cohort (n = 73) included only mild cases. Results are presented descriptively because the groups differ in epidemiological context and clinical composition. Ct, cycle threshold; IQR, interquartile range; N/A, not applicable. a Hospital stay was calculated only for severe pre-vaccination cases. b Deaths occurred only among severe pre-vaccination cases.

Clinical presentation differed across groups (**Figure 2A**). Pre-vaccination severe cases were characterized mainly by respiratory manifestations, particularly dyspnea and desaturation, whereas mild cases from both periods showed predominantly nonspecific symptoms. The interval between symptom onset and sample collection was shorter in the post-vaccination group than in pre-vaccination mild and severe cases (5 [3-6] and 5 [4-7] days, respectively). Ct values were lower in the post-vaccination group than in the pre-vaccination cohort, with only small differences between pre-vaccination mild and severe cases (**Table 1**).

**Figure 2:**
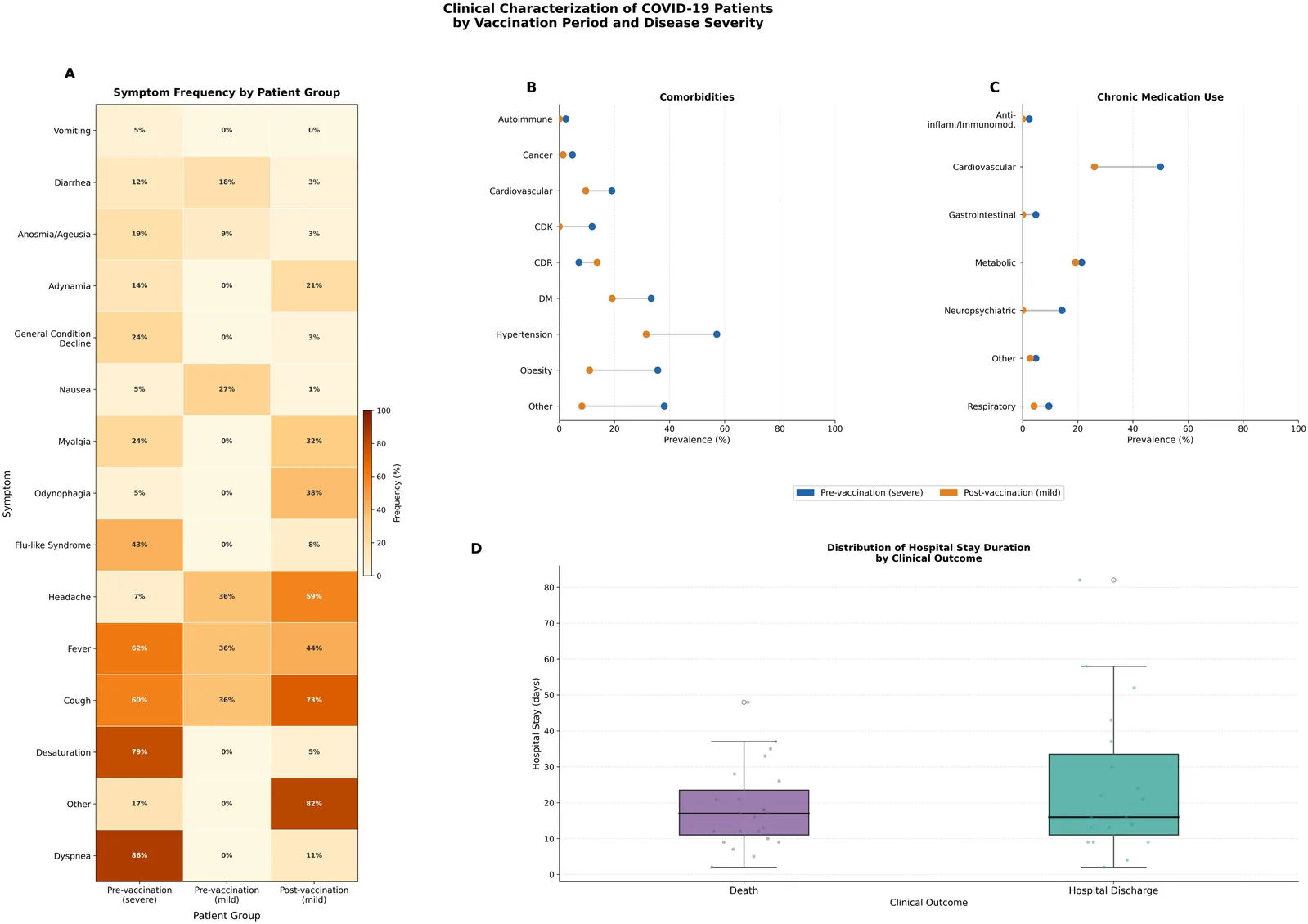
Clinical characterization of COVID-19 patients across pre- and post-vaccination periods. **(A)** Heatmap of symptom frequencies (%) in pre-vaccination severe (n = 42), pre-vaccination mild (n = 11), and post-vaccination mild (n = 73) groups. **(B)** Comorbidity prevalence (%) in pre-vaccination severe and post-vaccination mild groups. **(C)** Prevalence (%) of chronic medication use categories in the same groups; No chronic medication use was reported in the pre-vaccination mild group. Connecting lines are shown for visual comparison only and do not indicate paired observations. **(D)** Length of hospital stay (days) by outcome (death vs hospital discharge) among hospitalized pre-vaccination severe cases, shown as boxplots (median, interquartile range) with overlaid individual values. Panel **(B)** - CDR, chronic respiratory disease; CKD, chronic kidney disease; DM, diabetes mellitus. Panel **(C)** - Anti-inflam./Immunomod., anti-inflammatory and immunomodulatory drugs.

Comorbidities and chronic medication use also differed between groups (**Figures 2B, C**). Cardiometabolic conditions predominated among pre-vaccination severe cases, especially hypertension, obesity, and diabetes mellitus, whereas hypertension and diabetes remained the most frequent comorbidities in post-vaccination mild cases. No chronic medication use was reported in the pre-vaccination mild group. Among the groups with reported use, cardiovascular and metabolic drugs were the most frequent classes.

All severe pre-vaccination cases required hospitalization, with a median length of stay of 16.5 (10.5-27.5) days, 54.8% of these patients died (**Table 1**; **Figure 2D**). In contrast, all cases in the local post-vaccination cohort were mild, with no hospitalizations or deaths.

To contextualize these clinical differences, viral lineage composition was assessed in the same local cohort (**Figure 3**). The pre-vaccination period was dominated by pre-VOC lineages, mainly B.1.1.33 and B.1.1.28, whereas the post-vaccination period showed complete replacement by Omicron sublineages, predominantly BQ.1.1. Based on this distribution, subsequent analyses focused on the predominant lineage of each period, B.1.1.33 in the pre-vaccination phase and BQ.1.1 in the post-vaccination phase.

**Figure 3:**
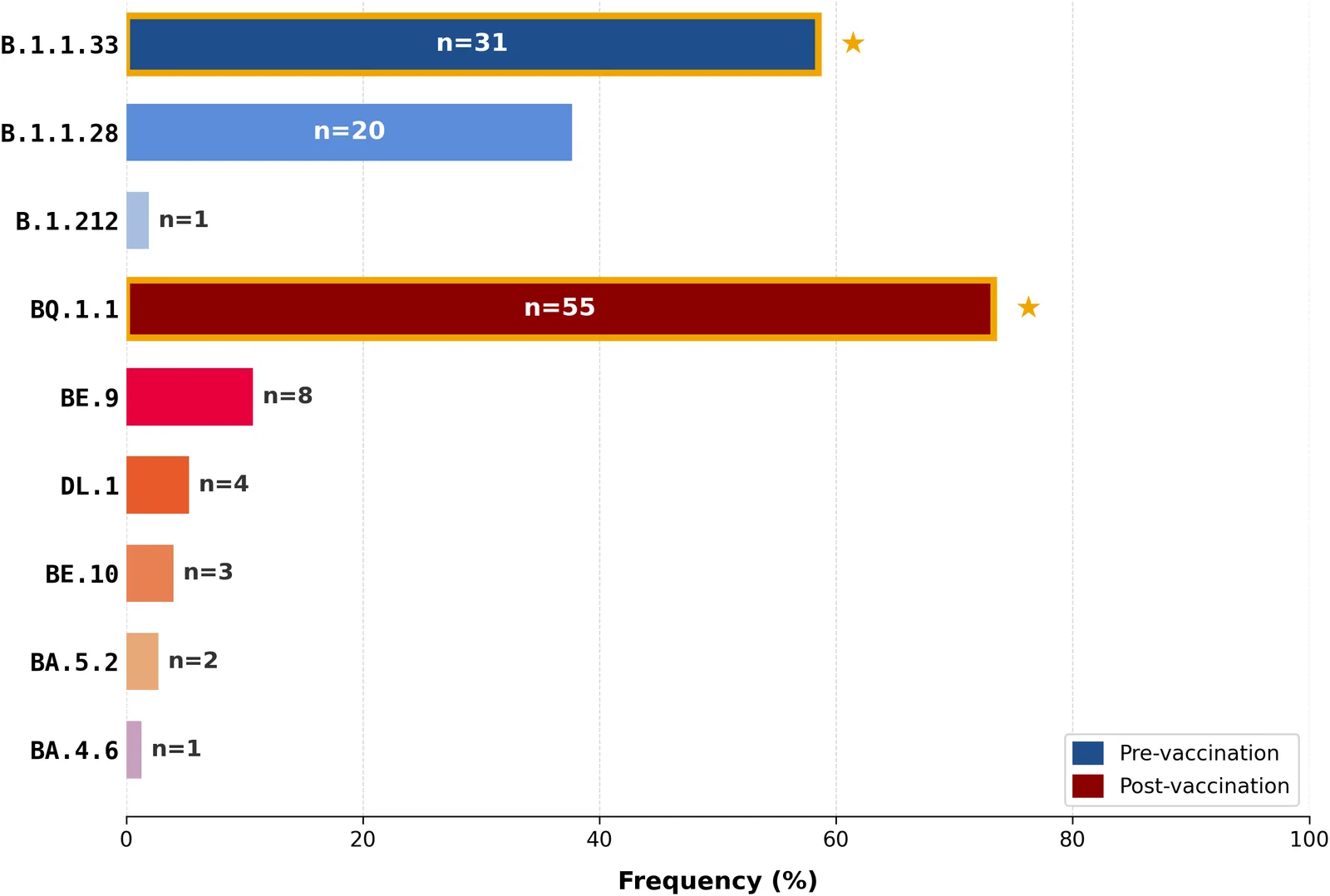
Distribution of SARS-CoV-2 lineages in the local cohort across pre- and post-vaccination periods. Horizontal bars show the proportion of locally sequenced samples assigned to each lineage in the pre-vaccination and post-vaccination periods. Numbers within or beside bars indicate lineage counts. Predominant lineages are marked with asterisks (*): B.1.1.33 (60.4%) in the pre-vaccination period and BQ.1.1 (75.3%) in the post-vaccination period.

### 3.2 Genomic analysis and mutational profile

Mutation-severity association analyses were performed in the expanded cohort, which combined local genomes with public GISAID sequences selected according to sequence quality and availability of clinical metadata. This approach was necessary because the local post-vaccination cohort included only mild cases. Accordingly, severity stratification in this section reflects the classification available in the source datasets and includes severe post-vaccination cases derived from the expanded cohort.

Comparison of the predominant lineages from each period, B.1.1.33 (pre-vaccination) and BQ.1.1 (post-vaccination), revealed distinct genome-wide mutational profiles. In the pre-vaccination B.1.1.33 group (n=401), 593 unique amino acid changes were identified, most of them occurring at very low frequency, consistent with a broad long-tail distribution and few near-fixed events. In contrast, the post-vaccination BQ.1.1 group (n=133) showed 388 unique amino acid changes, with a substantially higher proportion of recurrent mutations, particularly in Spike, indicating a more concentrated mutational profile.

To determine whether these differences also translated into within-period severity contrasts, mutation frequencies were stratified by clinical outcome and subsequently tested in adjusted models (**Figure 4**; **Table 2**). In the pre-vaccination B.1.1.33 cohort, severe cases were older than mild cases (median 65 years, IQR 51-76, vs 40 years, IQR 29-56; p < 0.001). In the adjusted logistic model, age remained independently associated with severity, whereas sex was not independently associated. Among eligible mutations, Q23K was more frequent in severe than in mild cases (11.0% vs 2.0%, among genomes with complete covariates), and remained associated with severity after adjustment for age and sex. Given its low frequency in mild cases, this estimate should be interpreted cautiously as an exploratory, hypothesis-generating signal. Accordingly, this signal was used to prioritize an antigenically located NTD change for structural analysis, rather than as evidence that Q23K increases disease severity. To assess whether alternative amino acid states occur at this position, all substitutions identified at Spike residue 23 were examined across the full expanded pre-vaccination cohort (n = 401), without applying the 5% prevalence threshold used in the initial mutation screening. Q23K was the only substitution detected at this site throughout the dataset, with no evidence of additional amino acid variants, thereby precluding comparative analyses at this position.

**Figure 4:**
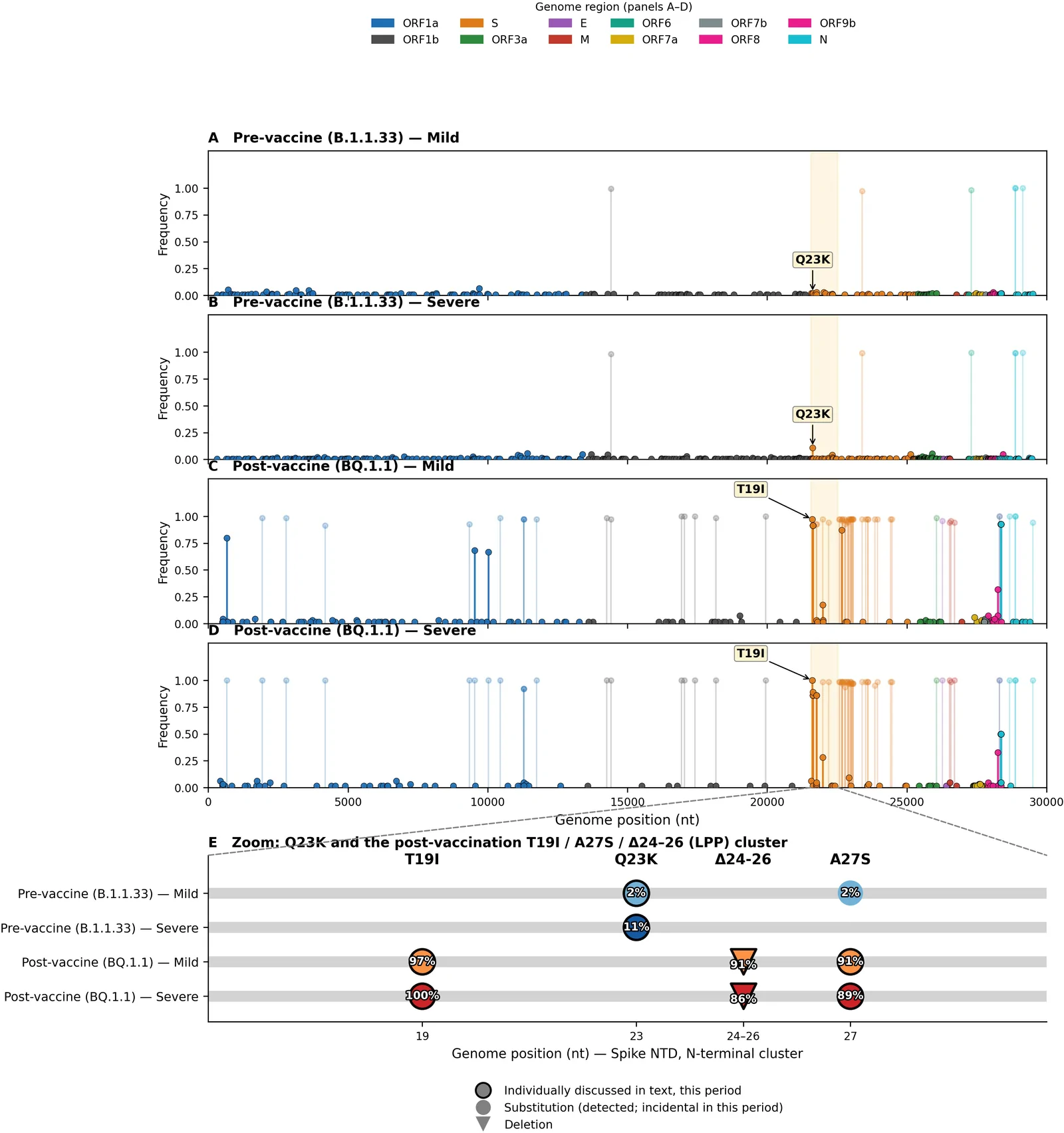
Genome-wide mutation frequencies stratified by disease severity within the pre-vaccination B.1.1.33 and post-vaccination BQ.1.1 cohorts. Genome-wide mutation frequencies mapped to the Wuhan-Hu-1 reference genome (x-axis; nucleotide coordinates) and stratified by disease severity within each cohort. Panels show pre-vaccination B.1.1.33 mild **(A)** and severe **(B)**, and post-vaccination BQ.1.1 mild **(C)** and severe **(D)**. Each lollipop represents a mutation, and its height indicates the proportion of genomes carrying that mutation within the corresponding subgroup (y-axis). Faded markers denote high-frequency lineage-defining variants (≥90%) displayed with reduced opacity for clarity. The shaded region corresponding to the Spike N-terminal domain (NTD; residues 14-320) is expanded in panel **(E)**. **(E)** Expanded view of the NTD N-terminal region (residues 14-46), highlighting Q23K and the T19I/A27S/Δ24-26 (LPP) cluster across all subgroups. Circles indicate substitutions and triangles indicate deletions. Black outlines mark mutations discussed in the main text (Q23K in pre-vaccination groups; T19I, A27S, and Δ24–26 in post-vaccination groups). Percentages indicate the proportion of genomes carrying each mutation within the corresponding subgroup.

**Table 2:** Association of demographic variables and selected viral mutations with disease severity in the expanded pre-vaccination (B.1.1.33) and post-vaccination (BQ.1.1) cohorts.

Variable		Mild cases	Severe cases	p-value	OR (95% CI)	p-value (OR)
Pre-vaccination (B.1.1.33) 153 Mild cases 248 Severe cases						
Age (years), median (IQR)		40 (29-56)	65 (51-76)	< 0.001*	1.82 (1.59-2.09)*	< 0.001
Sex, n (%)	Male	71 (46.4)	116 (46.8)	0.88	0.81 (0.50-1.30) #	0.379
	Female	82 (53.6)	131 (52.8)		–	–
	Unknown	0 (0.0)	1 (0.4)		–	–
Viral mutation, n (%)	Q23K	3/151 (2.0)	26/237 (11.0)	< 0.001*	6.94 (1.83-26.28)	0.004* (age+sex; n=388)
Post-vaccination (BQ.1.1) 69 Mild cases 64 Severe cases						
Age (years), median (IQR)		41 (31-55)	52 (36-74)	0.007*	1.73 (1.35-2.21)*	< 0.001
Sex, n (%)	Male	22 (31.9)	33 (51.6)	0.034*	1.97 (0.78-5.00) #	0.154
	Female	46 (66.7)	31 (48.4)			—
	Unknown	1 (1.4)	0 (0.0)			
Viral mutation, n (%) ‡	ORF9b:Δ27-29/N:Δ31-33	63/68 (92.6)	31/63 (49.2)	< 0.001*	0.038 (0.011-0.13)	< 0.001* (age+sex; n=131)

*p* < 0.05. ORs (95% CIs) were obtained from binary logistic regression models (outcome: 0 = mild; 1 = severe) adjusted for age and sex. Age was modeled per 10-year increment (age/10). # Sex OR = male vs female (female as the reference category); records classified as “Unknown” were excluded from sex comparisons and adjusted models. ORs for mutations/events = presence vs absence. Adjusted models were restricted to individuals with known age and sex (pre-vaccination: *n* = 388; post-vaccination: *n* = 131). Unadjusted *p* values: age by Mann-Whitney *U* test; sex by Chi-square test (male vs female); mutations by Fisher’s exact test. ORF9b:Δ27–29 and N:Δ31–33 represent the same nucleotide event in an overlapping reading-frame region and were therefore treated as a combined event. ‡ In the post-vaccination cohort, all severe cases were obtained from public GISAID genomes, whereas mild cases originated from both local sequencing (n=30) and GISAID (n=39); clinical outcome and sequencing source are therefore partially confounded. A sensitivity analysis restricted to GISAID-only samples (39 mild, 64 severe) yielded an age- and sex-adjusted OR of 0.08 (95% CI 0.02-0.27; p<0.001), consistent in direction and significance with the unstratified estimate. Because no locally sequenced severe cases exist, a fully crossed source × outcome comparison remains impossible, and residual heterogeneity across GISAID-submitting laboratories/pipelines cannot be excluded; the odds ratio shown is therefore reported for transparency and is not interpreted as a fully validated severity effect estimate.

In the post-vaccination BQ.1.1 cohort, severe cases were also older than mild cases (52 [36–74] vs 41 [31–55] years; p=0.007), and men were more frequent among severe cases in the univariate analysis (51.6% vs 31.9%; p=0.034). However, in the adjusted model, only age remained independently associated with severity, whereas sex did not.

The main mutational signal in this cohort was the overlapping deletion event annotated as ORF9b:Δ27-29/N:Δ31-33. This variant was more frequent in mild than in severe cases (92.6% vs 49.2%) and remained associated with lower odds of severity after adjustment (**Table 2**). During this period, all severe cases originated from public GISAID genomes, whereas mild cases were derived from both local sequencing (n = 30) and GISAID (n = 39), resulting in partial confounding between outcome and sequence source.

A sensitivity analysis restricted to GISAID-only samples (39 mild, 64 severe) showed that the association persisted (87.2% vs 50.0%; unadjusted OR = 6.80, 95% CI 2.36-19.61, Fisher’s exact p < 0.001; age- and sex-adjusted OR = 0.08, 95% CI 0.02-0.27, p < 0.001, using the same severe outcome convention as **Table 2**). This indicates that the signal is not solely attributable to the local-versus-GISAID split. Nevertheless, as public GISAID submissions originate from multiple laboratories and sequencing pipelines, residual source or pipeline heterogeneity cannot be excluded. In addition, this type of indel event is particularly susceptible to calling artifacts. Accordingly, this association is not interpreted as a validated candidate severity signal (see Discussion).

After Benjamini-Hochberg correction across all mutations meeting the 5% prevalence threshold within each cohort (7 pre-vaccination and 71 post-vaccination candidates), Q23K remained the only significant signal in the pre-vaccination dataset (q = 0.004). In the post-vaccination cohort, three additional ORF1a substitutions also remained significant (S135R, T3090I, and T3255I; all q ≤ 0.001), each present in 100% of severe cases and in 66-79% of mild cases. Given that these are near-fixed lineage-defining changes in BQ.1.1, the observed prevalence differences are more plausibly explained by variation in coverage or sequencing quality across submitting laboratories rather than by an effect on severity. These variants were therefore not carried forward (**Supplementary Table 3**).

Across periods, the post-vaccination lineage exhibited a cluster of NTD changes in Spike (T19I/A27S/Δ24–26, hereafter LPP) located adjacent to the Q23K position identified in the pre-vaccination lineage. None of these changes was individually associated with severity in the post-vaccination cohort (T19I, 97.1% in mild vs 100.0% in severe, p = 0.497; A27S, 91.2% vs 88.9%, p = 0.773; Δ24–26, 91.2% vs 85.7%, p = 0.414; **Supplementary Table 3**). Moreover, T19I and Δ24–26 are lineage-defining Omicron features that reached high frequency through global lineage expansion rather than selection within this cohort.

Accordingly, the LPP motif was not included in structural analyses based on any association with severity. Instead, it was examined because it represents recurrent sequence variation within the same NTD supersite neighborhood occupied by Q23K in the preceding period, an adjacency directly illustrated in **Figure 4E**. This topological continuity, rather than an epidemiological signal, underlies the rationale for the subsequent structural analysis of the NTD–*1-87* interface.

Because the harmonized severe/hospital-based category included hospitalization, ICU admission, mechanical ventilation, and death, we additionally tested whether the Q23K-severity association held under a stricter, unambiguous outcome. In the local cohort, severity is already defined by ICU admission (Section 2.1), so the 26 locally sequenced severe cases already met this stricter criterion. Within the GISAID-derived subgroup, the 222 severe cases were reclassified by reported clinical status, restricting the strict-severe group to the 122 cases labeled “Deceased”. The remaining 100 cases labeled only as hospitalized, inpatient, or unspecified were excluded from this comparison. In the resulting age- and sex-adjusted model (strict-severe [local ICU + GISAID deceased], n = 148, vs mild, n = 153), Q23K remained associated with severity (26/148, 17.6% vs 3/153, 2.0%; adjusted OR = 16.67, 95% CI 4.09-68.04, p = 0.0001), higher than the OR obtained under the broader severity definition. As with the primary estimate, the wide confidence interval (three mild carriers) still limits precision, and the finding remains exploratory.

Because Q23K co-occurred with other rare substitutions, its phylogenetic context was examined to determine whether severe and mild Q23K-positive cases segregated into distinct clonal lineages, which would suggest that the observed association with disease severity was driven by a single transmission cluster rather than a recurrent signal. Severe and mild Q23K-positive genomes were interspersed across multiple branches of the phylogenetic tree, with no evidence that either clinical outcome formed an exclusive or independent clade (**Figure 5A**). Geographic annotation of the tree indicated a marked concentration of Q23K-positive genomes among branches originating from Espírito Santo (ES), compared to genomes from other Brazilian states and from other countries. To quantify this pattern beyond visual inspection of the similarity-selected phylogenetic dataset, a systematic, non-similarity-based query of all B.1.1.33 genomes available in GISAID within the same collection period was performed (**Supplementary Methods S5.2**).

**Figure 5:**
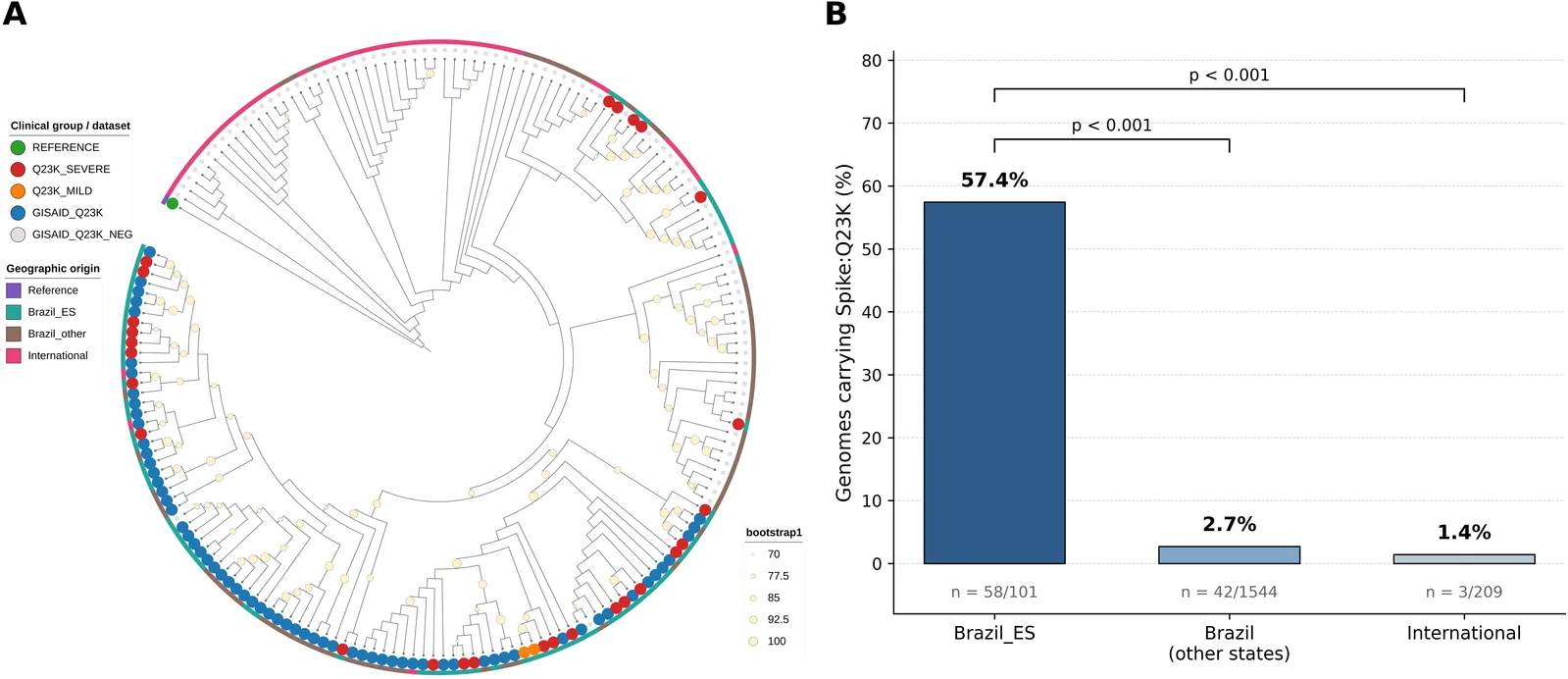
Phylogenetic context of SARS-CoV-2 sequences with the Q23K mutation and their clinical and geographic prevalence **(A)** Maximum-likelihood phylogenetic tree showing the distribution of genomes carrying the Spike Q23K mutation within the pre-vaccination B.1.1.33 dataset. Public GISAID sequences were included based on sequence similarity and overlapping collection dates (September 2020 to March 2021). Terminal nodes are annotated according to clinical group and dataset (Q23K-positive severe and mild cases, public GISAID Q23K-positive and Q23K-negative genomes, and the reference sequence). The outer ring indicates geographic origin (Espírito Santo, other Brazilian states, and international sequences). Bootstrap support values are shown for major clades. **(B)** Proportion of genomes carrying the Q23K mutation across geographic groups. Bars represent the percentage of Q23K-positive genomes within each group (Espírito Santo, other Brazilian states, and international sequences), with sample sizes indicated below each category. Statistical comparisons were performed using a two-sided Fisher’s exact test, with p-values indicated.

This analysis confirmed a substantially higher prevalence of Q23K in ES (57.4%; 58/101) compared to other Brazilian states (2.7%; 42/1,544) and international sequences (1.4%; 3/209), with differences remaining highly significant (two-sided Fisher’s exact test, p < 0.001 for both comparisons; **Figure 5B**).

### 3.3 Structural modeling and molecular dynamics

AlphaFold-derived confidence metrics were examined before molecular-dynamics analysis. The isolated NTD predictions showed overall pTM scores of 0.87 for WT, 0.85 for Q23K, and 0.84 for LPP; with per-residue confidence (pLDDT) mapped onto the structures in **Supplementary Figure 6**. The WT model showed high overall residue-level confidence, with a mean Cα pLDDT of 88.10 ± 12.78. Confidence was lower in the N3 loop, residues 141–156, which showed a mean pLDDT of 73.72 ± 5.09. The N5 loop, residues 246–260, presented markedly lower confidence, with a mean pLDDT of 51.36 ± 15.50 and a median of 44.57. Nine of the 15 N5 residues had pLDDT values below 50, indicating substantial uncertainty in the predicted local conformation of the central portion of this loop.

Structural modeling and molecular dynamics simulations of the NTD-*1-87* complex were performed for WT, Q23K, and LPP, based on the pre-vaccination association of Q23K with severity and on adjacent NTD changes observed post-vaccination. All models showed acceptable stereochemical quality, with similar Ramachandran profiles and ≤0.6% of residues in disallowed regions (**Supplementary Figure 2**).

Across the pooled 600-ns dataset (three independent 200-ns replicates per condition), the WT complex showed the greatest deviation and the largest radius of gyration and solvent-accessible surface area, whereas LPP adopted a more compact conformation with lower RMSD, Rg, and SASA; Q23K showed deviation comparable to WT, with smaller radius of gyration and solvent-accessible surface area (**Figures 1A, C, D**). In all systems, flexibility remained concentrated mainly in loop regions, indicating local rearrangements without altering the overall pattern of loop-enriched mobility (**Figure 1B**).

Convergence and inter-replicate reproducibility were assessed before pooling (**Supplementary Methods S7.1**; **Supplementary Tables 5**, **S6**). Rg and SASA were the most reproducible properties across the three independent replicates (CV = 0.3-4.2% and 0.8-2.6%, respectively), with cumulative means stabilizing within the first 20–30 ns and no systematic drift across subsequent 20-ns blocks. RMSD was the least reproducible (CV = 16.7-20.2%): each replicate was internally stable after approximately 50 ns, but the plateau values differed between replicates, most clearly in WT, where one replicate converged near 0.50 nm and the other two near 0.65-0.71 nm. Intermediate inter-replicate heterogeneity was observed for ΔG (CV = 3.5-10.7%) and BSA (CV = 9.0-21.1%). Per-atom RMSF profiles were strongly correlated between replicates in all three systems (Pearson r = 0.77-0.93; mean absolute error 0.025-0.045 nm), with LPP showing the lowest inter-replicate agreement of the three.

Predicted interface energetics showed the same overall trend (**Figure 1E**). Relative to WT, both Q23K and LPP displayed more favorable ΔG values, with ΔΔG of -0.49 and -0.72 kcal/mol, respectively. Predicted Kd values followed the same direction but varied markedly between replicates (CV = 32-89%; **Supplementary Table 5**) and are therefore reported for completeness only and not interpreted. These magnitudes are within the reported error of the PRODIGY predictor (RMSE ≈ 1.89 kcal/mol) and should therefore not be interpreted as quantitative affinity differences, only the direction and consistency of the trend across the frame-wise distribution are considered informative here. In parallel, buried surface area decreased from WT to Q23K and LPP (**Figure 1F**). Thus, the mutant complexes combined lower SASA and Rg with smaller BSA, alongside a small shift toward more favorable predicted binding energies. The global effect size for ΔG was negligible (ϵ² = 0.066), and the WT replicate means ranged from −8.74 to −10.72 kcal/mol (**Supplementary Table 5**), a spread comparable to the difference between WT and the mutants; predicted binding energy is therefore not interpreted as differing between systems. This combination of smaller BSA with more favorable predicted ΔG may partly reflect the sensitivity of the scoring function to contact chemistry, so the interpretation of contact redistribution rests primarily on the trajectory-derived contact-persistence analysis.

Frame-wise distributions of SASA, ΔG, and BSA, pooled across the three independent replicates per condition, differed significantly between WT and both mutant systems. Kruskal-Wallis tests indicated significant overall differences among WT, Q23K, and LPP for ΔG (H(2) = 1198.73, p < 0.0001, ϵ² = 0.066), SASA (H(2) = 2292.35, p < 0.0001, ϵ² = 0.127), and BSA (H(2) = 10139.11, p < 0.0001, ϵ² = 0.563). In Dunn’s *post hoc* comparisons using WT as the reference group, both Q23K and LPP differed significantly from WT for all three parameters after Benjamini-Hochberg adjustment (all adjusted p < 0.0001). Cliff’s δ values for Q23K and LPP relative to WT were -0.20 and -0.34 for ΔG, -0.38 and -0.46 for SASA, and -0.48 and -0.94 for BSA.

Contact persistence analysis further supported this gradient (**Table 3**). WT showed a broader and less consolidated interface at the residue-pair level, with low median contact occupancy and only one ultra-stable interaction, despite recurrent engagement of selected NTD residues along the trajectory. Q23K increased the number of contacts and their persistence, whereas LPP, despite fewer total contacts, showed the most anchored interaction regime, with the highest median occupancy and the largest number of ultra-stable and hyper-stable contacts. Representative anchors included ASP253-GLY:B:411 in WT, TRP152-PRO:B:414 in Q23K, and LYS147-GLU:B:379 plus GLY252-ASP:B:423 in LPP. Residue-time interaction maps further illustrated this reorganization, with Q23K (**Supplementary Figure 4**) and especially LPP (**Supplementary Figure 5**) showing more sustained occupancy across key supersite-associated NTD residues than WT (**Supplementary Figure 3**). At the residue level, supersite hotspots remained relevant across systems, but engaged alternative partners and interaction types in the mutants, particularly in N3 and N5 regions (**Supplementary Table 4**; see **Supplementary Figure 7** for the local structural context of the mutated residues at the *1-87* interface).

**Table 3:** Comparative summary of NTD-1–87 interface organization and interaction hotspots in WT, Q23K, and LPP models.

Model	Panel A- Global Interface Metrics (200 ns)					Panel B- Interaction Hotspots (Top-3 per System)		
	Total Contacts	Median Occupancy (%)	Ultra-stable (≥90%)	Hyper-stable (≥99%)	Continuous Anchors	Primary Anchors (occupancy %)	Antibody Hubs (hotspot score)	NTD Hubs (hotspot score)
WT	131	0.5	1	0	0	- ASP253-GLY:B:411 (H-bond)- 92.0% - LYS147-GLU:B:338 (salt)- 65.5% - TYR145-PRO:B:413 (hydrof.)- 62.0%	- GLU338 - 104.0 - GLY411 - 94.8 - PRO413 - 87.0	- LYS147- 106.4 - ASP253- 92.9 - TYR145- 79.7
Q23K	161	1.8	1	1	1	- TRP152-PRO:B:414 (hydrof.)- 100.0% - TYR248-GLU:B:338 (H-bond)- 78.5% - TRP152-PRO:B:412 (hydrof.)- 74.4%	- PRO:B:414- 203.8 - GLU:B:338- 131.0 - PRO:B:413- 118.3	- TYR248- 284.2 - LYS147- 218.2 - TRP152- 209.9
T19I/A27S/Δ24-26 (LPP)	93	8.7	12	5	2	- LYS147-GLU:B:379 (salt)- 100.0% - GLY252-ASP:B:423 (H-bond)- 100.0% - LYS147-ILE:B:337 (H-bond)- 99.8%	- ASP:B:423- 205.4 - PHE:B:358- 184.4 - SER:B:492- 172.9	- LYS147- 497.3 - TYR248- 399.4 - PRO251- 286.0

Continuous anchors were defined as contacts maintained without interruption throughout the analyzed trajectory, representing a stricter category than ultra-stable contacts, which were classified by occupancy threshold only. Hotspot score denotes a cumulative trajectory-derived metric of residue participation in persistent interface contacts and should be interpreted comparatively within this analysis, not as an absolute energetic measure. Chains B and C correspond to the heavy and light chains of the 1-87 Fab in PDB 7L2D, respectively.

## 4 Discussion

This study integrated clinical-epidemiological and genomic data by combining local sequencing from the State of Espírito Santo in Brazil with publicly available GISAID genomes to investigate how the transition between the pre-vaccination and post-vaccination phases was reflected in clinical severity and in the mutational architecture of SARS-CoV-2. The analysis was structured from both within-period and across-period perspectives, without assuming direct comparability between these settings. During the pre-vaccination period, B.1.1.33 circulated in a context of low accumulated immunity and greater reliance on non-pharmaceutical interventions (De Souza Santos et al., 2021; Sonabend et al., 2021), whereas the post-vaccination period, dominated by BQ.1.1, unfolded under an immune landscape reshaped by vaccination, prior infection, and expansion of Omicron sublineages associated with immune evasion (Ito et al., 2023). This framing is essential for interpreting the findings, because mass vaccination in Brazil progressively changed the population susceptibility profile, with reductions in average severity and shifts in mortality patterns over the course of the pandemic (Li et al., 2024; Santos et al., 2022).

In the local dataset, the clinical asymmetry between periods was pronounced: the pre-vaccination phase concentrated hospitalized cases and a higher proportion of severe disease, whereas the post-vaccination phase included only mild cases. This contrast is consistent with the broader epidemiological shift observed over the course of the pandemic and with the transition toward globally milder clinical profiles after immune accumulation. However, it also reflects differences in sampling strategy. Accordingly, the post-vaccination set should not be interpreted as representing the clinical spectrum of Omicron overall (Relan et al., 2023; Santos et al., 2022). In agreement with previous cohorts, severe pre-vaccination cases were older, a finding later corroborated in the expanded cohort and consistent with the well-established role of age as a determinant of worse outcome. The higher proportion of men in this group should be interpreted more cautiously because this pattern may also be influenced by sample composition (Santos et al., 2022; Zeiser et al., 2021).

Lower Ct values in the post-vaccination period do not contradict this framework, as their interpretation is limited by pre-analytical and analytical variability and by the shorter interval between symptom onset and sampling, which is consistent with earlier capture of infection and with the well-recognized inconsistency of the association between Ct and severity in the literature (Dananché et al., 2022; Frediani et al., 2024; Rao et al., 2020). Likewise, the predominance of cardiometabolic comorbidities and lower respiratory tract involvement among severe pre-vaccination cases is in line with the profile reported in hospital-based cohorts, whereas the milder presentation and predominance of upper respiratory symptoms in the post-vaccination group parallels the clinical pattern more frequently described in Omicron-associated waves (Paravidino et al., 2022; Vihta et al., 2023). Taken together, these findings indicate that the observed severity profile reflected the interaction between baseline clinical status, immune context, and timing of sampling, rather than an isolated effect of the period itself.

Consistent with the global and national pattern of successive variant replacement throughout the pandemic, the local circulation observed here showed a transition from early lineages in the pre-vaccination period to Omicron sublineages in the post-vaccination phase (Carabelli et al., 2023; Naveca et al., 2021). The predominance of B.1.1.33 and B.1.1.28 in the pre-vaccination period agrees with the Brazilian scenario of 2020 and early 2021, before variants of concern became established, whereas the predominance of BA.5 descendants, particularly BQ.1.1, parallels the pattern described for the second half of 2022, when sublineages carrying additional Spike changes rapidly co-circulated and replaced closely related variants (de Souza et al., 2025; Giovanetti et al., 2022; Ito et al., 2023). The greater intravariant diversity observed in the post-vaccination dataset is likewise consistent with the more recent landscape of convergent evolution and rapid local shifts in dominance among Omicron descendants (Markov et al., 2023). Interpretation of severity-associated signals in the expanded cohort nevertheless requires caution, because the dataset combined local and public sequences with heterogeneous metadata and demographic differences between mild and severe cases. For this reason, the mutational signals should be interpreted as context-dependent associations rather than direct causal inferences. Even so, the frequency landscape supports the view that the two periods reflected distinct evolutionary regimes: the pre-vaccination phase showed a long tail of rare mutations, compatible with drift, transmission structure, and still-heterogeneous selection, whereas the post-vaccination phase showed a greater concentration of recurrent events, especially in Spike, in agreement with stronger selection under high population immunity (Ito et al., 2023; Naveca et al., 2021).

Within these distinct evolutionary backgrounds, two mutational signals were particularly notable. In the pre-vaccination period, Q23K in the Spike NTD was enriched in severe cases and remained associated with severity after adjustment for age and sex. However, because it was rare among mild cases, the magnitude of this association should be interpreted cautiously as an exploratory, hypothesis-generating finding.Its location in the NTD is nevertheless biologically relevant, as this region contains a major antigenic supersite and represents a recurrent site of substitutions and deletions associated with altered recognition by neutralizing antibodies (McCallum et al., 2021; Cerutti et al., 2021). Importantly, this relevance is antigenic rather than receptor-mediated: the NTD contains a distinct antibody supersite, whereas ACE2 recognition is mediated by the RBD of Spike (Cerutti et al., 2021; Wrapp et al., 2020). The functional interpretation of Q23K and the adjacent NTD changes therefore concerns recognition by NTD-directed antibodies rather than direct receptor binding, providing the rationale for focusing the structural analysis on the antibody interface. A more indirect allosteric contribution to receptor binding cannot be formally excluded but lies beyond the scope of the present NTD-focused model.

Severe and mild Q23K-positive genomes were interspersed across the phylogeny rather than forming exclusive, outcome-segregated subclades (**Figure 5A**), reducing the likelihood that the observed association with disease severity is driven by a single transmission cluster. This pattern is consistent with the geographic distribution of Q23K. Both the phylogenetic annotation and a systematic, non-similarity-based GISAID query of B.1.1.33 genomes from the same collection window (**Supplementary Methods S5.2**) indicate a marked concentration of Q23K in Espírito Santo (57.4%; 58/101), compared to other Brazilian states (2.7%) and international sequences (1.4%; **Figure 5B**). Taken together, these findings indicate that Q23K represents a recurrent, regionally frequent variant in ES, not restricted to a single clinical outcome or transmission chain, rather than a broadly distributed pattern or the result of a single clustered outbreak. Because the systematic geographic analysis was conducted independently of the sequences included in the discovery dataset, this regional enrichment is unlikely to reflect ascertainment bias in the phylogenetic sampling. However, the data remain consistent with a local transmission cluster or founder effect specific to Espírito Santo during this period, which cannot be further resolved here.

In the post-vaccination period, the frequency difference observed for ORF9b:Δ27-29/N:Δ31–33 warrants careful interpretation. As already noted, this comparison is partially confounded by sequencing source, all severe cases were obtained from GISAID, but mild cases were drawn from both local sequencing and GISAID, which allowed a sensitivity analysis restricted to GISAID-only samples. This analysis showed that the association persisted (age- and sex-adjusted OR = 0.08, 95% CI 0.02-0.27, p<0.001), indicating that the local-vs-GISAID split alone does not account for the signal. This locus also corresponds to a single-nucleotide deletion within an overlapping reading frame, a class of variation known to be particularly sensitive to sequencing depth, assembly strategies, and indel-calling approaches (Jungreis et al., 2021). Moreover, public GISAID submissions themselves originate from heterogeneous laboratories and pipelines, so residual source/pipeline-related artifact cannot be excluded even within the GISAID-only stratum. We also did not assess whether this deletion represents a sublineage-defining feature within BQ.1.1, which could further contribute to the observed distribution. Given these limitations, ORF9b:Δ27-29/N:Δ31–33 is not interpreted as a validated marker of disease severity. Instead, it is retained as an exploratory finding and as a representative example of the potential for source–outcome confounding in genomic surveillance analyses that integrate heterogeneous sequencing datasets (Han et al., 2021). Taken together, these contrasts supported the structural stage as a conformational plausibility test: although Q23K did not persist into the post-vaccination period, adjacent NTD changes, such as LPP, became recurrent, suggesting continued remodeling of the same antigenic neighborhood rather than persistence of a single mutation. Notably, no viral change in the post-vaccination period was retained as a candidate marker of greater disease severity. The signals detected either pointed toward non-severe disease or were consistent with sequencing-source confounding. This pattern is consistent with the milder clinical profile observed in later periods being influenced substantially by accumulated population immunity, rather than indicating viral evolution toward increased or decreased virulence (Carabelli et al., 2023; Markov et al., 2023).

In this context, the *1-87* antibody should be viewed as a biologically grounded structural probe rather than as a representation of the full anti-NTD humoral response. Although anti-NTD immunity is polyclonal, *1-87* captures a recurrent mode of supersite recognition supported by experimental structural data, making it suitable for testing the plausibility of conformational remodeling in this region (Lok, 2021; Wibmer et al., 2021). This choice is further supported by experimental evidence showing that Omicron-associated changes in the NTD supersite, including T19I and Δ24-26, are linked to reduced neutralization by NTD-directed antibodies (Cao et al., 2022; Wang et al., 2022).

Against this background, structural modeling combined with molecular dynamics simulations indicates that the Q23K mutation, and more markedly LPP, are associated with a reorganization of the NTD-*1-87* interaction profile. In comparison to the wild-type (WT), these variants favor interaction modes that are less diffuse and exhibit increased conformational stability, consistent with more compact and persistent complexes. This is supported by reduced radius of gyration (Rg) and solvent-accessible surface area (SASA), with reduced RMSD in LPP, alongside a small shift toward more favorable predicted ΔG/Kd values whose global effect size was negligible (ϵ² = 0.066) and which was not consistent across replicates (**Supplementary Table 5**). Notably, the reduction in buried surface area was the largest and most reproducible effect observed (ϵ² = 0.563; Cliff’s δ = −0.48 and −0.94 for Q23K and LPP), with the direction preserved in all three replicates, suggesting a shift from surface-driven interactions toward binding energetics dominated by structurally organized hotspots and sustained contact networks, as previously described in protein-protein interaction studies (Bogan and Thorn, 1998; Vangone and Bonvin, 2015). This shift is corroborated primarily by the trajectory-derived contact-persistence analysis (**Table 3**), given that the ΔG/BSA covariation alone may partly reflect the sensitivity of the scoring function to contact chemistry.

Importantly, the absence of any reduction in predicted binding affinity in the context of a single Fab interacting with an isolated NTD monomer should not be interpreted as evidence of antibody escape, if anything, it indicates preserved structural compatibility. In other words, the variant NTDs remained at least as compatible with the antibody as the wild type, showing no loss of binding rather than the weaker recognition that simple antibody escape would predict. A plausible explanation for this apparent discrepancy lies in the architectural features of the NTD supersite, which is largely defined by flexible, glycan-proximal loops, particularly within the N3 and N5 regions (Cerutti et al., 2021; McCallum et al., 2021; Lok, 2021). Subtle rearrangements in these exposed segments may alter epitope geometry, solvent accessibility, or glycan shielding when considered in the context of the fully glycosylated Spike trimer, without necessarily impacting the intrinsic affinity of a pre-formed Fab-NTD complex (Watanabe et al., 2020).

It is worth noting that glycans were not explicitly modeled in the present simulations, and any mechanistic interpretation involving glycan-mediated effects remains speculative pending experimental validation (Watanabe et al., 2020). Rather than indicating loss of binding compatibility, the data support a redistribution of intermolecular contacts within the supersite (Barnes et al., 2020). The functional implications of such remodeling, however, cannot be inferred from a monomeric, single-antibody, pre-formed complex model. In particular, neutralization potency is not solely determined by monomeric binding affinity, but also depends on additional factors such as the angle of antibody approach relative to the Spike trimer, the achievable binding stoichiometry across protomers, and the dynamic interplay with ACE2 engagement and Spike conformational transitions (e.g., RBD up/down states) (Wrapp et al., 2020; Barnes et al., 2020). These aspects, along with binding kinetics and avidity effects, are not captured in the present modeling framework (Klein and Bjorkman, 2010).

The extent of this contact-level reorganization appears larger than expected given the limited number of substituted or deleted residues, particularly when considered alongside the ΔG estimates, which fall within the reported error range of the PRODIGY predictor (Vangone and Bonvin, 2015) and are therefore not used to support quantitative affinity differences. In this framework, recognition by anti-NTD antibodies depends strongly on loop conformation, such that point substitutions or short deletions within or adjacent to these regions can locally reshape geometry and electrostatics without disrupting the overall NTD fold, consistent with the preserved stereochemical quality of all models (≤0.6% of residues in disallowed Ramachandran regions; **Supplementary Figure 2**). Accordingly, Q23K and LPP do not indicate loss of structural compatibility with *1-87* once the complex is formed, but instead redistribute and consolidate specific intermolecular contacts. This interpretation carries a modeling caveat that should be stated rather than left implicit: the N3 and N5 loops on which the contact reorganization is centered are also the lowest-confidence regions of the AlphaFold3 predictions (**Supplementary Figure 6**). The specific residue-pair assignments reported in **Table 3** should therefore be read as the behavior of these models, not as established contacts.

None of this rules out antibody escape either. The results point to a reorganization of the supersite contact network and a shift in the mode of recognition, without directly addressing functional neutralization. NTD alterations associated with variants such as Omicron are well established to reduce antibody neutralization (Cao et al., 2022; Wang et al., 2022), likely through mechanisms that lie outside the scope of the present model, including altered association kinetics and epitope accessibility. Structural reorganization and immune escape are therefore not mutually exclusive, and a locally more consolidated interface may still correspond to reduced neutralization in the context of the intact Spike trimer and the polyclonal antibody response.

The limitations of the analytical design of this study should be considered when interpreting these findings. The local cohort was small and clinically unbalanced, particularly in the pre-vaccination mild subgroup, whereas the local post-vaccination dataset included only mild cases, limiting representativeness of the clinical spectrum and precluding symmetrical local comparisons between periods. In the post-vaccination period specifically, the absence of locally sequenced severe cases means that outcome and sequence source cannot be fully disentangled through a local-vs-GISAID stratified design. Beyond sequencing source, post-vaccination immunity was itself highly heterogeneous across individuals, differing in vaccine type, time since the last dose, and history of prior infection, which further limits inference from this period. For this reason, post-vaccination severity signals are treated as non-inferential, and the LPP cluster is carried forward on structural grounds alone. However, because mild cases included both local (n=30) and GISAID (n=39) samples, a sensitivity analysis restricted to GISAID-only samples was feasible (Results 3.2, **Table 3**) and showed the association persisted, although it cannot rule out residual heterogeneity across GISAID-submitting laboratories and pipelines. Additionally, publicly available GISAID metadata for the post-vaccination expanded cohort were limited to sex, age, clinical status at collection, and geographic location; Ct values and symptom counts were not available for the GISAID-derived severe subgroup, precluding alternative severity-independent outcome models (e.g., Ct value, symptom count) as a way to disentangle outcome from sequence source in this period. Expanding this analysis to additional post-vaccination time windows or independent cohorts was not feasible within the scope of the present study and is noted as a direction for future work. Restricting the analysis to genomes with ≥90% coverage favored higher-quality sequences, but may have introduced selection bias, with lower representation of samples with low viral load or greater fragmentation. Some clinical variables were also incomplete or asymmetrically available across subgroups within the local cohort, and differences in the interval between symptom onset and sampling also limit direct comparability of Ct values and symptom profiles between the local pre- and post-vaccination datasets. In the expanded cohort, heterogeneity in clinical metadata, severity definitions, sequence origin, and annotation quality may have influenced the magnitude of the observed associations, particularly for rare events or those sensitive to coverage and indel calling. Accordingly, mutational signals should be interpreted as context-dependent and hypothesis-generating rather than as evidence of direct clinical causality. Finally, the structural stage of this study was conducted as a conformational plausibility test in a single modeled NTD-*1-87* complex, which is biologically relevant, but does not capture the full diversity of the anti-NTD humoral response, and was not functionally validated. No ELISA, SPR/BLI or neutralization assay was performed, and no experimental binding measurement supports the modeled interface, all interface conclusions are therefore computational. Several further limitations apply to this stage. The NTD chain was modeled as a monomer and the complex was assembled by rigid superposition onto the experimentally resolved 7L2D pose rather than co-folded, so interface geometry was not independently optimized and the conformational fit at the antibody-antigen interface was not energetically validated. In addition, the simulations assess a pre-formed complex and therefore inform on complex stability rather than on association kinetics or on epitope accessibility in the glycosylated trimer, and a single antibody probe cannot exclude that conformational compaction occludes epitopes for antibodies approaching the supersite at different angles.

## 5 Conclusion

This study shows that the transition between the pre-vaccination and post-vaccination phases of the COVID-19 pandemic involved not only lineage replacement, but also reconfiguration of the mutational repertoire in antigenically relevant regions of Spike. Using within- and across-period comparisons, mutational signals differed between cohorts, most notably Q23K in the pre-vaccination period and a deletion event at the overlapping ORF9b/N locus in the post-vaccination period. Although the latter association persisted in a sensitivity analysis restricted to GISAID samples, it is presented as an illustration of the potential for source–outcome confounding in genomic surveillance, rather than as a validated severity marker. Q23K, the strongest pre-vaccination signal, is best understood as a recurrent, regionally concentrated substitution within an antigenically relevant region of Spike rather than as a determinant of clinical severity. Structural modeling and molecular dynamics of the NTD–1–87 complex indicated that alterations in distinct lineages converged on the same antigenic neighborhood of the NTD, with structural reorganization of the antibody–antigen interface relative to WT, providing conformational plausibility for hypotheses of altered immune recognition in this domain. Taken together, these findings support the integration of contextualized genomic surveillance and structural analysis as a strategy to monitor antigenically relevant changes in SARS-CoV-2 circulation.

## Data Availability

The datasets presented in this study can be found in online repositories. The names of the repository/repositories and accession number(s) can be found in the article/**Supplementary Material**.
