# Supplementary material for: Antigenic remodeling of the SARS-CoV-2 Spike N-terminal domain links sequence variability to structural reorganization of a neutralizing antibody supersite
Source: Front Cell Infect Microbiol. 2026 Sep 14;16:1924817. doi: 10.3389/fcimb.2026.1924817 (PMC13616644; doi:10.3389/fcimb.2026.1924817)
Supplement: Supplementary file 1 [file DataSheet1.pdf]

Supplementary Material

Supplementary Methods

S1. Sample sources and period-specific cohort composition

In the pre-vaccination period, severe cases were obtained from Hospital Estadual Jayme dos Santos Neves (hospital cohort), whereas the mild subgroup comprised outpatient samples from primary care units in Vila Velha, Espírito Santo, Brazil.

In the post-vaccination period, samples collected for routine diagnosis at LACEN/ES (including referrals from Hospital Jayme dos Santos Neves and primary care units in Vitória, Espírito Santo, Brazil) were retrospectively included, with clinical and demographic data retrieved from electronic medical records.

Only mild cases were available in the local post-vaccination cohort. Therefore, severe post-vaccination cases were incorporated exclusively in the expanded-cohort stage using public GISAID genomes with available clinical metadata (*patient status*), after category harmonization.

S2. RNA extraction and RT-qPCR

RNA extraction was performed using the Biogene Viral DNA/RNA Extraction Kit (Bioclin, Brazil), according to the manufacturer's instructions.

Real-time RT-qPCR was performed using 1-Step RT-qPCR Master Mix (Thermo Fisher Scientific, Waltham, MA, USA) on an Applied Biosystems platform (Carlsbad, CA, USA). Primers and hydrolysis probes targeting the N1 and N2 regions of the nucleocapsid gene, together with RNase P as internal control, were used following CDC recommendations.

Negative and positive controls (2019-nCoV RUO Kit, Integrated DNA Technologies, IDT, Iowa, USA) were included in all runs. Samples with Ct < 27 were selected for whole-genome sequencing.

Although LACEN/ES samples had been previously tested for routine diagnosis, all samples included in this study were retested under the same protocol. For Ct-based analyses, the N2 target generated in the study laboratory was used as the reference measure.

S3. ONT amplification, library preparation, and sequencing

Selected-sample RNA was reverse-transcribed using LunaScript RT SuperMix and amplified in two primer pools using the Midnight protocol (EXP-MRT001), generating overlapping ~1200-bp amplicons spanning the SARS-CoV-2 genome. Amplicon pools were quantified using a dsDNA HS kit, and samples with adequate concentration proceeded to library preparation.

To improve coverage in Omicron samples, the following additional primers were added (Supplementary Table S1):

Supplementary Table S1. Additional primers incorporated into the 1200 bp amplicon scheme to improve genome coverage in Omicron samples.

Pool	Primer name	Sequence (5'→3')
**Pool 1**	Pool1_1200_23_Left_Omicron	ACTTTAGAGTTCAACCAACAGAATCT
**Pool 1**	Pool1_1200_21_Right_Omicron	GTGTATGATTGAGTTCTGGTTGTAAG
**Pool 2**	Pool2_1200_22_Right_Omicron	AACAGATGCAAATTTGGTGGCG
**Pool 2**	Pool2_1200_24_Left_Omicron	GCTGAATATGTCAACAACTCATATGA
**Pool 2**	Pool2_1200_28_Left_Omicron	TTTGTGCTTTTTAGCCTTTCTGTT

Amplicons from both pools were combined and purified with Ampure XP beads (1× volume).

Libraries were prepared using the ONT Rapid Barcoding Kit 96 (SQK-RBK-110.96) and sequenced on MinION R9.4.1 flow cells (FLO-MIN106) using a MinION Mk1C device (Oxford Nanopore Technologies, ONT).

Raw data were acquired with MinKNOW, and high-accuracy basecalling and initial quality control were performed using Guppy v6.0.1.

S3.4 Consensus generation and lineage assignment

Consensus sequences were generated using the ARTIC Network SARS-CoV-2 workflow (read processing, mapping, and consensus calling). Quality control, coverage assessment (whole genome and target regions), and clade/lineage assignment were performed in Nextclade.

S4. Expanded cohort eligibility and clinical metadata harmonization

The expanded cohort integrated locally generated genomes and public GISAID genomes selected according to the predominant lineage in each period (B.1.1.33 for pre-vaccination and BQ.1.1 for post-vaccination).

Inclusion criteria were: (i) ≥90% whole-genome coverage; (ii) ≥90% Spike coverage; and (iii) for public genomes, availability of clinical metadata (*patient status*).

GISAID *patient status* entries were curated and harmonized into a binary severity variable before statistical analyses (Supplementary Table S2). Two harmonized categories were used: mild/non-severe and severe/hospital-based.

The mild/non-severe category included labels indicating no hospitalization or low clinical severity (eg, *asymptomatic*, *mild*, *no clinical signs*, *outpatient*, and *paucisymptomatic*). The severe/hospital-based category included labels indicating hospitalization or critical disease (eg, *hospitalized*, *inpatient*, *ICU*, *intubated*, *ventilation*), as well as *death/deceased* records.

Labels that were ambiguous, non-clinical, or insufficient to define severity according to the harmonization rules were treated as missing and excluded from severity-based regression analyses. Harmonization was applied consistently across the expanded cohorts.

Supplementary Table S2. Rule-based harmonization of original GISAID *patient status* labels into binary severity categories used in expanded-cohort analyses.

Harmonized category	Original GISAID	Included in severity analysis
**Mild / Severe**	**Patient status label**	**Yes/No**
**Mild / Non-severe**	Asymptomatic	Yes
	Mild	Yes
	No clinical signs	Yes
	Outpatient	Yes
	Paucisymptomatic	Yes
**Severe / Hospital-based**	Hospitalized	Yes
	Inpatient	Yes
	Death	Yes

	Deceased	Yes
	ICU	Yes
	Intubated	Yes
	Ventilation	Yes
**Missing / Excluded**	Unknown / not provided / N/A	No
	Any ambiguous/non-clinical label (released; Discharge)	No

Original GISAID patient status entries were harmonized into a binary severity variable (mild/non-severe vs severe/hospital-based) before statistical analyses. Harmonization was rule-based and applied consistently within each period-specific expanded cohort. Labels that were ambiguous, non-clinical, or insufficient to define severity were treated as missing and excluded from severity-based analyses. In the public-metadata harmonization, the severe/hospital-based category included hospitalized/inpatient records and markers of critical disease (eg, ICU admission, intubation/ventilation), as well as deceased cases.

S5.1 Phylogenetic dataset selection and annotation parameters (S:Q23K analysis)

For the S:Q23K phylogenetic context analysis, public GISAID genomes were selected by similarity using Audacity Instant Search. The following parameters were used: max search = 10; min quality = 0.9; min matches = 40; max matches = 100. The retrieval was restricted to the same collection interval as the B.1.1.33 study group (September 2020 to March 2021).

The consensus tree was annotated in iTOL using predefined terminal categories: Q23K*SEVERE; Q23K*MILD; GISAID*Q23K; GISAID*Q23K*NEG; REFERENCE. Geographic origin was also annotated for visual contextualization.*

S5.2 Systematic estimation of S:Q23K prevalence by geographic origin

To estimate the prevalence of Spike:Q23K among B.1.1.33 genomes independently of the similarity-based selection used for the phylogenetic dataset (Supplementary Methods S5.1), a systematic GISAID query was performed, restricted to lineage B.1.1.33, the same collection window as the pre-vaccination cohort (September 2020 to March 2021), and filtered for Complete and High-coverage genomes, without restriction by clinical metadata availability. The proportion of

genomes carrying S:Q23K was calculated for Espírito Santo (ES), other Brazilian states, and internationally. Group proportions were compared using two-sided Fisher's exact tests.

S6. Structural modeling and molecular dynamics

S6.1 Structural modeling workflow

The reference Spike–antibody complex was obtained from the RCSB PDB (7L2D). The modeled Spike fragment corresponded to the NTD plus adjacent region (residues S:14-320, Wuhan-Hu-1 numbering).

The NTD chain was modeled using AlphaFold3. Starting from the WT model, two variant models were generated: Q23K (pre-vaccination representative) and T19I/A27S/Δ24-26 (LPP) (post-vaccination representative).

Other NTD substitutions were not included in this reduced structural model. Structural alignment of each NTD model to the reference complex was performed in PyMOL, and NTD-*1-87* complexes were assembled by structural superposition. Stereochemical quality was assessed using PROCHECK (SAVe v6.1).

S6.2 MD system preparation and simulation settings

Molecular dynamics simulations were performed in GROMACS v2024.2 using the CHARMM36 force field. Each complex was solvated in a dodecahedral box with TIP3P water, with a minimum distance of 3.0 nm between any protein atom and the box edge.

Systems were neutralized and adjusted to 0.15 M Na^+ and Cl^-. Energy minimization was performed using steepest descent until the maximum force was < 1000 kJ/(mol·nm).

After energy minimization, each system underwent sequential NVT and NPT equilibration (0.1 ns each) with a 2 fs integration step, using a v-rescale thermostat at 300 K and a c-rescale barostat at 1 bar, before the production simulations.

Bonds involving hydrogen atoms were constrained using LINCS. Long-range electrostatics were treated with Particle Mesh Ewald (PME), using a 1.2-nm cutoff.

Production runs were performed for 100,000,000 steps (2-fs integration step), in three independent 200-ns replicas per condition, totaling 600 ns per condition.

S6.3 Trajectory and interface analyses

Trajectories were visualized in PyMOL and analyzed using XMGRACE v5.1.25. Trajectory analysis included root mean square deviation (RMSD), root mean square fluctuation (RMSF), solvent-accessible surface area (SASA), and radius of gyration (Rg). Predicted interfacial interactions (hydrogen bonds, salt bridges, π-cation, π-stacking, and hydrophobic contacts) were identified using PLIP. Interaction outputs were organized into interaction maps using in-house Python scripts, the hotspot score was computed as the sum of occupancies (%) of all contacts involving a given residue across the analysed trajectory.

S6.4 Binding-affinity and interface-area estimates

For frame-wise comparative affinity estimates, each trajectory frame was preprocessed with PDBFixer (including addition of missing atoms when needed) and submitted to PRODIGY to estimate binding free energy (ΔG) and dissociation constant (Kd). Buried surface area (BSA) was calculated for all frames using FreeSASA.

S7. Statistical implementation details

Binary logistic regression models used harmonized clinical severity as the outcome, coded as 0 = mild/non-severe and 1 = severe/hospital-based. Binary predictors, including mutation status, were coded as 0 = absent and 1 = present. Accordingly, OR > 1 indicates higher odds of severe/hospital-based classification, whereas OR < 1 indicates lower odds (Supplementary Table S3).

Mutation screening was performed separately within each period-specific expanded cohort, using only records with known age, sex, and severity. Candidate mutations were required to show prevalence ≥ 5% before unadjusted association testing. Only mutations retaining signal after this screening stage were entered into multivariable logistic regression models adjusted for age and sex. Age was modeled per 10-year increase.

For molecular dynamics-derived parameters (SASA, BSA, and ΔG), normality was assessed using the Shapiro-Wilk test in GraphPad Prism v9.0.0.121. Given non-normality, and because WT, Q23K, and LPP were simulated as independent systems (frame-wise measurements not paired across conditions), comparisons were performed using the Kruskal-Wallis test followed by Dunn's post hoc test with Benjamini-Hochberg adjustment, with the WT complex used as the reference group. Effect magnitude was quantified using epsilon squared (ε^2) for the omnibus test and Cliff's delta (δ) for pairwise comparisons against WT.

Software used: IBM SPSS Statistics for Windows v21.0 (IBM Corp., Armonk, NY, USA) for inferential analyses; Python 3.10 (pandas, numpy, matplotlib) for descriptive analyses and plots; and R 4.3.2 (stats, ggplot2, rcompanion) for descriptive analyses and plots. Effect sizes (e2 and Cliff's d) were computed in R 4.3.2 using the rcompanion and effsize packages.

S7.1 Convergence and reproducibility assessment

For RMSD, Rg, SASA, ΔG, and BSA, convergence within each 200-ns replicate was assessed by computing the cumulative (running) mean over time and by dividing each trajectory into ten 20-ns blocks, from which block-wise means and standard deviations were obtained. The last 100 ns (100-200 ns) of each replicate was defined as the production phase. Reproducibility across the three independent replicates per condition was quantified as the coefficient of variation (CV% = 100 × SD/|mean|) of the production-phase means across replicates. RMSF, which has no time axis, was instead assessed for inter-replicate reproducibility using the Pearson correlation coefficient and mean absolute error between per-atom profiles of each replicate pair. Results are reported in Supplementary Tables S5-S6.

Supplementary Table S3. Univariate screening of SARS-CoV-2 mutations with frequency ≥5% for association with disease severity in the expanded pre-vaccination (B.1.1.33) and post-vaccination (BQ.1.1) cohorts.

Cohort	Mutation	Eligible prevalence n/N (%)	Mild n/N (%)	Severe n/N (%)	Unadjusted p (Fisher)	Passes unadjusted	Adjusted OR (95% CI)	Adjusted p	FDR q-value (BH)

						screening (p<0.05)			
**Pre-vaccination**	N:I292T	388/388 (100.0%)	151/151 (100.0%)	236/236 (100.0%)	1	No	–	–	1
**Pre-vaccination**	N:R203K	387/388 (99.7%)	151/151 (100.0%)	235/236 (99.6%)	1	No	–	–	1
**Pre-vaccination**	N:G204R	387/388 (99.7%)	151/151 (100.0%)	235/236 (99.6%)	1	No	–	–	1
**Pre-vaccination**	ORF6:I33T	385/388 (99.2%)	148/151 (98.0%)	236/236 (100.0%)	0.059	No	–	–	0.205
**Pre-vaccination**	ORF1b:P314L	384/388 (99.0%)	150/151 (99.3%)	233/236 (98.7%)	1	No	–	–	1
**Pre-vaccination**	S:D614G	383/388 (98.5%)	147/151 (97.4%)	235/237 (99.2%)	0.213	No	–	–	0.498
**Pre-vaccination**	S:Q23K	29/388 (7.5%)	3/151 (2.0%)	26/237 (11.0%)	<0.001	**Yes**	6.94 (1.83-26.28)	0.004	0.004
**Post-vaccination**	ORF9b:Δ27-29 / N:Δ31-33	94/131 (71.8%)	63/68 (92.6%)	31/63 (49.2%)	<0.001	**Yes**	0.038 (0.01-0.13)	<0.001	<0.001
**Post-vaccination**	N:G204R	131/131 (100.0%)	68/68 (100.0%)	63/63 (100.0%)	1	No	–	–	1
**Post-vaccination**	N:R203K	131/131 (100.0%)	68/68 (100.0%)	63/63 (100.0%)	1	No	–	–	1
**Post-vaccination**	N:E136D	131/131 (100.0%)	68/68 (100.0%)	63/63 (100.0%)	1	No	–	–	1
**Post-vaccination**	N:P13L	131/131 (100.0%)	68/68 (100.0%)	63/63 (100.0%)	1	No	–	–	1
**Post-vaccination**	ORF1b:T2163I	131/131 (100.0%)	68/68 (100.0%)	63/63 (100.0%)	1	No	–	–	1
**Post-vaccination**	ORF1b:R1315 C	131/131 (100.0%)	68/68 (100.0%)	63/63 (100.0%)	1	No	–	–	1
**Post-vaccination**	ORF9b:P10F	131/131 (100.0%)	68/68 (100.0%)	63/63 (100.0%)	1	No	–	–	1
**Post-vaccination**	ORF1b:N1191 S	131/131 (100.0%)	68/68 (100.0%)	63/63 (100.0%)	1	No	–	–	1
**Post-vaccination**	ORF1b:M1156 I	131/131 (100.0%)	68/68 (100.0%)	63/63 (100.0%)	1	No	–	–	1
**Post-vaccination**	ORF1b:Y264H	130/131 (99.2%)	67/68 (98.5%)	63/63 (100.0%)	1	No	–	–	1
**Post-vaccination**	ORF1a:T842I	130/131 (99.2%)	67/68 (98.5%)	63/63 (100.0%)	1	No	–	–	1
**Post-vaccination**	ORF3a:T223I	130/131 (99.2%)	67/68 (98.5%)	63/63 (100.0%)	1	No	–	–	1
**Post-vaccination**	ORF1b:I1566V	130/131 (99.2%)	67/68 (98.5%)	63/63 (100.0%)	1	No	–	–	1
**Post-vaccination**	ORF1a:Q556K	130/131 (99.2%)	67/68 (98.5%)	63/63 (100.0%)	1	No	–	–	1
**Post-vaccination**	ORF1a:P3395 H	130/131 (99.2%)	67/68 (98.5%)	63/63 (100.0%)	1	No	–	–	1
**Post-vaccination**	ORF1b:P314L	129/131 (98.5%)	66/68 (97.1%)	63/63 (100.0%)	0.497	No	–	–	1
**Post-vaccination**	S:T19I	129/131 (98.5%)	66/68 (97.1%)	63/63 (100.0%)	0.497	No	–	–	1
**Post-vaccination**	ORF1a:L3829 F	129/131 (98.5%)	66/68 (97.1%)	63/63 (100.0%)	0.497	No	–	–	1
**Post-vaccination**	S:D614G	128/131 (97.7%)	66/68 (97.1%)	62/63 (98.4%)	1	No	–	–	1
**Post-vaccination**	S:G142D	128/131 (97.7%)	66/68 (97.1%)	62/63 (98.4%)	1	No	–	–	1
**Post-vaccination**	S:T376A	128/131 (97.7%)	66/68 (97.1%)	62/63 (98.4%)	1	No	–	–	1
**Post-vaccination**	S:D405N	128/131 (97.7%)	66/68 (97.1%)	62/63 (98.4%)	1	No	–	–	1
**Post-vaccination**	S:S373P	128/131 (97.7%)	66/68 (97.1%)	62/63 (98.4%)	1	No	–	–	1
**Post-vaccination**	S:H655Y	128/131 (97.7%)	66/68 (97.1%)	62/63 (98.4%)	1	No	–	–	1
**Post-vaccination**	S:R346T	128/131 (97.7%)	66/68 (97.1%)	62/63 (98.4%)	1	No	–	–	1

**Post-vaccination**	S:G339D	128/131 (97.7%)	66/68 (97.1%)	62/63 (98.4%)	1	No	–	–	1
**Post-vaccination**	S:N679K	128/131 (97.7%)	66/68 (97.1%)	62/63 (98.4%)	1	No	–	–	1
**Post-vaccination**	S:P681H	128/131 (97.7%)	66/68 (97.1%)	62/63 (98.4%)	1	No	–	–	1
**Post-vaccination**	S:N969K	128/131 (97.7%)	66/68 (97.1%)	62/63 (98.4%)	1	No	–	–	1
**Post-vaccination**	S:D796Y	128/131 (97.7%)	66/68 (97.1%)	62/63 (98.4%)	1	No	–	–	1
**Post-vaccination**	S:Q954H	128/131 (97.7%)	66/68 (97.1%)	62/63 (98.4%)	1	No	–	–	1
**Post-vaccination**	S:S477N	128/131 (97.7%)	66/68 (97.1%)	62/63 (98.4%)	1	No	–	–	1
**Post-vaccination**	E:T9I	128/131 (97.7%)	65/68 (95.6%)	63/63 (100.0%)	0.245	No	–	–	1
**Post-vaccination**	M:D3N	128/131 (97.7%)	65/68 (95.6%)	63/63 (100.0%)	0.245	No	–	–	1
**Post-vaccination**	S:T478K	128/131 (97.7%)	66/68 (97.1%)	62/63 (98.4%)	1	No	–	–	1
**Post-vaccination**	S:N460K	127/131 (96.9%)	66/68 (97.1%)	61/63 (96.8%)	1	No	–	–	1
**Post-vaccination**	S:N501Y	127/131 (96.9%)	66/68 (97.1%)	61/63 (96.8%)	1	No	–	–	1
**Post-vaccination**	S:E484A	127/131 (96.9%)	66/68 (97.1%)	61/63 (96.8%)	1	No	–	–	1
**Post-vaccination**	S:L452R	127/131 (96.9%)	66/68 (97.1%)	61/63 (96.8%)	1	No	–	–	1
**Post-vaccination**	S:K444T	127/131 (96.9%)	65/68 (95.6%)	62/63 (98.4%)	0.62	No	–	–	1
**Post-vaccination**	N:S413R	127/131 (96.9%)	64/68 (94.1%)	63/63 (100.0%)	0.12	No	–	–	0.949
**Post-vaccination**	S:K417N	127/131 (96.9%)	65/68 (95.6%)	62/63 (98.4%)	0.62	No	–	–	1
**Post-vaccination**	S:Y505H	127/131 (96.9%)	66/68 (97.1%)	61/63 (96.8%)	1	No	–	–	1
**Post-vaccination**	S:Q498R	127/131 (96.9%)	66/68 (97.1%)	61/63 (96.8%)	1	No	–	–	1
**Post-vaccination**	M:Q19E	127/131 (96.9%)	65/68 (95.6%)	62/63 (98.4%)	0.62	No	–	–	1
**Post-vaccination**	M:A63T	127/131 (96.9%)	64/68 (94.1%)	63/63 (100.0%)	0.12	No	–	–	0.949
**Post-vaccination**	S:F486V	127/131 (96.9%)	66/68 (97.1%)	61/63 (96.8%)	1	No	–	–	1
**Post-vaccination**	S:N764K	126/131 (96.2%)	66/68 (97.1%)	60/63 (95.2%)	0.671	No	–	–	1
**Post-vaccination**	S:S375F	126/131 (96.2%)	65/68 (95.6%)	61/63 (96.8%)	1	No	–	–	1
**Post-vaccination**	S:N440K	126/131 (96.2%)	64/68 (94.1%)	62/63 (98.4%)	0.367	No	–	–	1
**Post-vaccination**	S:V213G	126/131 (96.2%)	64/68 (94.1%)	62/63 (98.4%)	0.367	No	–	–	1
**Post-vaccination**	ORF1a:L3027F	126/131 (96.2%)	63/68 (92.6%)	63/63 (100.0%)	0.059	No	–	–	0.595
**Post-vaccination**	ORF1a:G1307S	125/131 (95.4%)	62/68 (91.2%)	63/63 (100.0%)	0.028	**Yes**	–	–	0.336
**Post-vaccination**	ORF1a:S3675del	124/131 (94.7%)	66/68 (97.1%)	58/63 (92.1%)	0.26	No	–	–	1
**Post-vaccination**	ORF1a:F3677del	124/131 (94.7%)	66/68 (97.1%)	58/63 (92.1%)	0.26	No	–	–	1
**Post-vaccination**	ORF1a:G3676del	124/131 (94.7%)	66/68 (97.1%)	58/63 (92.1%)	0.26	No	–	–	1
**Post-vaccination**	S:R408S	122/131 (93.1%)	63/68 (92.6%)	59/63 (93.7%)	1	No	–	–	1
**Post-vaccination**	S:S371F	121/131 (92.4%)	59/68 (86.8%)	62/63 (98.4%)	0.018	**Yes**	5.857 (0.692-49.600)	0.105	0.253
**Post-vaccination**	S:A27S	118/131 (90.1%)	62/68 (91.2%)	56/63 (88.9%)	0.773	No	–	–	1

**Post-vaccination**	S:V70del	117/131 (89.3%)	63/68 (92.6%)	54/63 (85.7%)	0.261	No	–	–	1
**Post-vaccination**	S:H69del	117/131 (89.3%)	63/68 (92.6%)	54/63 (85.7%)	0.261	No	–	–	1
**Post-vaccination**	ORF1a:S135R	117/131 (89.3%)	54/68 (79.4%)	63/63 (100.0%)	<0.001	**Yes**	26.05 (3.28-3368.96)	<0.001	0.001
**Post-vaccination**	S:L24del	116/131 (88.5%)	62/68 (91.2%)	54/63 (85.7%)	0.414	No	–	–	1
**Post-vaccination**	S:P25del	116/131 (88.5%)	62/68 (91.2%)	54/63 (85.7%)	0.414	No	–	–	1
**Post-vaccination**	S:P26del	116/131 (88.5%)	62/68 (91.2%)	54/63 (85.7%)	0.414	No	–	–	1
**Post-vaccination**	ORF1a:T3090I	109/131 (83.2%)	46/68 (67.6%)	63/63 (100.0%)	<0.001	**Yes**	41.77 (5.42-5374.26)	<0.001	<0.001
**Post-vaccination**	ORF1a:T3255I	108/131 (82.4%)	45/68 (66.2%)	63/63 (100.0%)	<0.001	**Yes**	43.33 (5.61-5576.64)	<0.001	<0.001
**Post-vaccination**	ORF8:I121del	42/131 (32.1%)	22/68 (32.4%)	20/63 (31.7%)	1	No	–	–	1
**Post-vaccination**	S:Y144del	29/131 (22.1%)	12/68 (17.6%)	17/63 (27.0%)	0.214	No	–	–	1
**Post-vaccination**	ORF8:F6S	8/131 (6.1%)	5/68 (7.4%)	3/63 (4.8%)	0.72	No	–	–	1

Mutations/events with ≥5% overall prevalence were screened for association with disease severity (mild vs. severe) using Fisher's exact test. Variants passing unadjusted screening ($p < 0.05$) were entered into logistic regression adjusted for age and sex. Adjusted OR (95% CI) and adjusted p-values are reported where models converged. Rows highlighted in yellow indicate statistically significant associations after logistic regression adjustment (adjusted $p < 0.05$). Abbreviations: OR, odds ratio; CI, confidence interval; del, deletion. Benjamini-Hochberg false discovery rate (FDR) q-values were calculated separately within each cohort across all mutations/events meeting the ≥5% prevalence screening threshold, to formally account for multiple testing. For events showing quasi-complete separation under standard maximum-likelihood logistic regression, Firth's penalized logistic regression was used instead to obtain finite, interpretable adjusted OR, 95% CI, and p-values.Four post-vaccination events remained significant after adjustment (ORF9b:Δ27–29/N:Δ31–33, ORF1a:S135R, ORF1a:T3090I, and ORF1a:T3255I); the three ORF1a events showed complete or nearcomplete fixation within GISAID samples of both severity groups (consistent with a GISAID-specific lineage signature rather than a severity association), and ORF9b:Δ27–29/N:Δ31–33 showed marked enrichment, consistent with the partial clinical outcome × sequencing source confounding already described (Methods 2.1, Results 3.2, Discussion, Limitations) rather than a fully independent association with disease severity.

Supplementary Tables

Supplementary Tables S1–S3 are presented within the Supplementary Methods, at the point of their first citation.

Supplementary Table S4. NTD-*1-87* hotspot residues and contacts across WT, Q23K and T19I/A27S/Δ24-26 models.

Residue	Loop	WT Contact (%)	Q23K Contact (%)	T19I+A27S+Δ24-26/LPP Contact (%)
**VAL143**	**N3**	NA	VAL143-PRO:B:414 (Hydrof.; 58.62)<br>VAL143-PRO:B:413 (Hydrof.; 14.09)	VAL143-PRO:B:413 (Hydrof.; 0.65)
**TYR144**	**N3**	TYR144-GLU:B:338 (H-bond; 30.93)<br>TYR144-ILE:B:337 (Hydrof.; 11.29)	TYR144-ILE:B:337 (Hydrof.; 12.39)<br>TYR144-GLU:B:338 (H-bond; 8.95)	TYR144-ILE:B:337 (Hydrof.; 53.22)<br>TYR144-GLU:B:338 (Hydrof.; 42.68)
**TYR145**	**N3**	TYR145-PRO:B:413 (Hydrof.; 61.92)<br>TYR145-PRO:B:414 (Hydrof.; 16.69)	TYR145-THR:B:416 (H-bond; 60.32)<br>TYR145-PRO:B:413 (Hydrof.; 55.12)<br>TYR145-VAL:B:409 (Hydrof.; 44.18)	TYR145-VAL:B:409 (Hydrof.; 94.05)<br>TYR145-ALA:B:408 (Hydrof.; 70.51)
**LYS147**	**N3**	LYS147-GLU:B:338 (Salt; 65.52)<br>LYS147-PRO:B:413 (Hydrof.; 15.19)	LYS147-ILE:B:337 (H-bond; 50.32)<br>LYS147-ALA:B:363 (Hydrof; 25.94)<br>LYS147-GLU:B:379 (salt; 23.79)	LYS147-GLU:B:379 (Salt; 100.00)<br>LYS147-ILE:B:337 (H-bond; 99.80)<br>LYS147-PHE:B:358 (π-cation; 94.05)<br>LYS147-LEU:B:336 (H-bond; 91.90)<br>LYS147-PHE:B:358 (Hydrof.; 90.30)
**LYS150**	**N3**	LYS150-GLU:B:361 (Salt; 8.90)<br>LYS150-PRO:B:360 (H-bond; 1.25)	LYS150-ASP:B:362 (Salt; 52.97)<br>LYS150-GLU:B:361 (H-bond; 37.13)<br>LYS150-GLU:B:361 (Salt; 36.38)	LYS150-GLU:B:361 (H-bond; 76.71)<br>LYS150-ALA:B:363 (Hydrof.; 43.73)
**TRP152**	**N3**	TRP152-PRO:B:413 (Hydrof.; 7.30)<br>TRP152-PRO:B:414 (Hydrof.; 4.10)	TRP152-PRO:B:414 (Hydrof.; 100.00)<br>TRP152-PRO:B:412 (Hydrof.; 74.36)	TRP152-PRO:B:413 (Hydrof.; 66.07)

Residue	Loop	WT Contact (%)	Q23K Contact (%)	T19I+A27S+Δ24-26/LPP Contact (%)
			TRP152-PRO:B:413 (Hydrof.; 33.23)	TRP152-VAL:B:409 (Hydrof.; 33.83)<br>TRP152-THR:B:416 (H-bond; 23.74)<br>TRP152-THR:B:416 (Hydrof.; 23.29)
**ARG246**	**N5**	ARG246-ILE:B:337 (Hydrof.; 3.05)<br>ARG246-GLU:B:338 (Salt; 2.50)	ARG246-GLU:B:338 (Salt; 23.84)<br>ARG246-TYR:B:334 (H-bond; 6.20)	ARG246-GLU:B:338 (Salt; 95.10)<br>ARG246-TYR:B:334 (H-bond; 26.49)<br>ARG246-TYR:B:334 (π-cation; 24.14)
**TYR248**	**N5**	TYR248-TYR:B:334 (Hydrof.; 20.54)<br>TYR248-VAL:B:409 (Hydrof.; 7.75)	TYR248-GLU:B:338 (H-bond; 78.51)<br>TYR248-TYR:B:334 (Hydrof.; 57.97)<br>TYR248-THR:B:335 (H-bond; 43.28)<br>TYR248-TYR:B:334 (π-stack; 29.09)<br>TYR248-VAL:B:409 (Hydrof.; 28.19)	TYR248-ASP:B:423 (H-bond; 99.70)<br>TYR248-ILE:B:407 (Hydrof.; 93.80)<br>TYR248-ILE:B:407 (H-bond; 56.02)<br>TYR248-VAL:B:309 (Hydrof.; 49.23)<br>TYR248-TYR:B:334 (Hydrof.; 38.28)<br>TYR248-LEU:B:339 (Hydrof.; 34.18)
**PRO251**	**N5**	PRO251-PRO:B:412 (Hydrof.; 0.80)<br>PRO251-THR:B:416 (Hydrof.; 0.10)	PRO251-SER:C:492 (H-bond; 7.65)<br>PRO251-VAL:B:424 (Hydrof.; 6.15)	PRO251-SER:C:492 (H-bond; 99.50)<br>PRO251-TYR:B:419 (Hydrof.; 67.07)<br>PRO251-TYR:C:485 (Hydrof.; 59.42)<br>PRO251-LEU:C:482 (Hydrof.; 26.79)
**GLY252**	**N5**	GLY252-SER:B:415 (H-bond; 4.40)<br>GLY252-ILE:B:410 (H-bond; 1.50)	GLY252-SER:C:492 (H-bond; 4.00)<br>GLY252-TYR:C:485 (H-bond; 2.85)	GLY252-ASP:B:423 (H-bond; 100.00)<br>GLY252-GLN:B:308 (H-bond; 2.55)
**ASP253**	**N5**	ASP253-GLY:B:411 (H-bond; 91.95)<br>ASP253-ILE:B:410 (H-bond; 0.85)	ASP253-SER:C:492 (H-bond; 4.55)<br>ASP253-GLY:C:493 (H-bond; 2.75)	ASP253-SER:C:492 (H-bond; 67.87)<br>ASP253-GLN:B:308 (H-bond; 35.23)
**SER254**	**N5**	SER254-GLN:B:308 (H-bond; 19.29)<br>SER254-VAL:B:409 (H-bond; 1.75)	SER254-SER:C:492 (H-bond; 1.25)	SER254-GLN:B:308 (H-bond; 48.33)<br>SER254-VAL:B:309 (H-bond; 13.54)

Occupancy (%) was defined as the proportion of molecular dynamics frames in which a given NTD-*1-87* contact was detected (occupancy = N_frames/2,001 × 100). Values report occupancies over the full trajectory. For each

NTD residue, we list the main contacts observed in each system (including lower-occupancy contacts when they represent alternative pairings at key supersite residues). N3 (141–156) and N5 (246–260) refer to loop regions comprising the NTD antigenic supersite. Abbreviations: H-bond, Hydrogen bond; Hydrof., Hydrophobic; salt, Salt Bridge.

Supplementary Table S5. Reproducibility of RMSD, Rg, SASA, ΔG, BSA, and Kd across the three independent 200-ns replicates per condition (production-phase means, 100-200 ns) in WT, Q23K, and LPP. Kd is reported in micromolar (µM) for readability. CV% = 100 x SD/|mean| across replicates.

Metric	Variant	Replicate 1	Replicate 2	Replicate 3	Mean ± SD	CV%
**RMSD (nm)**	WT	0.692	0.709	0.516	**0.639 ± 0.106**	**16.7%**
**RMSD (nm)**	Q23K	0.596	0.787	0.540	**0.641 ± 0.129**	**20.2%**
**RMSD (nm)**	LPP	0.476	0.362	0.349	**0.396 ± 0.070**	**17.7%**
**Rg (nm)**	WT	3.345	3.233	3.073	**3.217 ± 0.137**	4.2%
**Rg (nm)**	Q23K	3.182	3.165	3.175	**3.174 ± 0.008**	0.3%
**Rg (nm)**	LPP	3.145	3.196	3.172	**3.171 ± 0.025**	0.8%
**SASA (nm²)**	WT	274.18	267.23	260.42	**267.28 ± 6.88**	2.6%
**SASA (nm²)**	Q23K	264.07	262.65	260.05	**262.26 ± 2.04**	0.8%
**SASA (nm²)**	LPP	261.70	265.82	254.27	**260.59 ± 5.86**	2.2%
**ΔG (kcal/mol)**	WT	−8.74	−10.72	−10.40	**−9.95 ± 1.06**	10.7%
**ΔG (kcal/mol)**	Q23K	−10.18	−10.25	−11.41	**−10.61 ± 0.69**	6.5%
**ΔG (kcal/mol)**	LPP	−10.47	−10.60	−11.17	**−10.75 ± 0.37**	3.5%
**BSA (nm²)**	WT	37.83	27.82	25.85	**30.50 ± 6.42**	**21.1%**
**BSA (nm²)**	Q23K	29.47	25.01	22.93	**25.80 ± 3.34**	13.0%
**BSA (nm²)**	LPP	23.63	21.78	19.75	**21.72 ± 1.94**	9.0%
**Kd (µM)**	WT	511.5	100.9	145.0	**252.5 ± 225.4**	**89.3%**
**Kd (µM)**	Q23K	177.8	139.4	54.9	**124.0 ± 62.9**	**50.7%**
**Kd (µM)**	LPP	114.3	97.5	58.0	**89.9 ± 28.9**	**32.2%**

Supplementary Table S6. Pairwise reproducibility of the per-atom RMSF profile between independent replicates (Pearson's r and mean absolute error, MAE) in WT, Q23K, and LPP.

Variant	Replicate pair	Pearson's r	MAE (nm)
**WT**	1–2	0.888	0.043
**WT**	1–3	0.898	0.045
**WT**	2–3	0.935	0.038
**Q23K**	1–2	0.862	0.027
**Q23K**	1–3	0.874	0.027
**Q23K**	2–3	0.871	0.025

**LPP**	1–2	0.833	0.035
**LPP**	1–3	0.773	0.037
**LPP**	2–3	0.823	0.032

Supplementary Figures

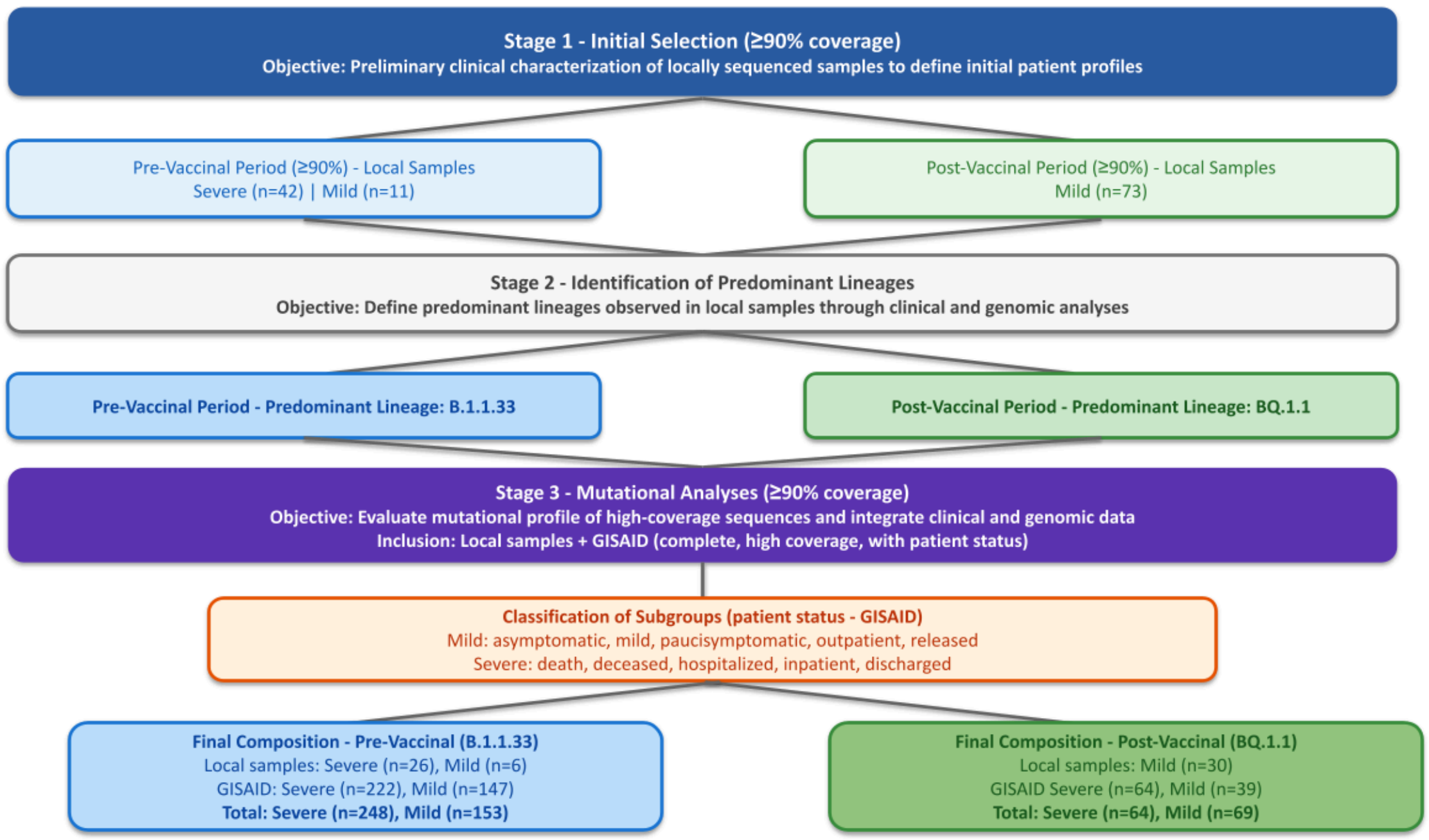

Supplementary Figure S1. Flowchart of sample selection and final dataset composition for clinical and genomic analyses.

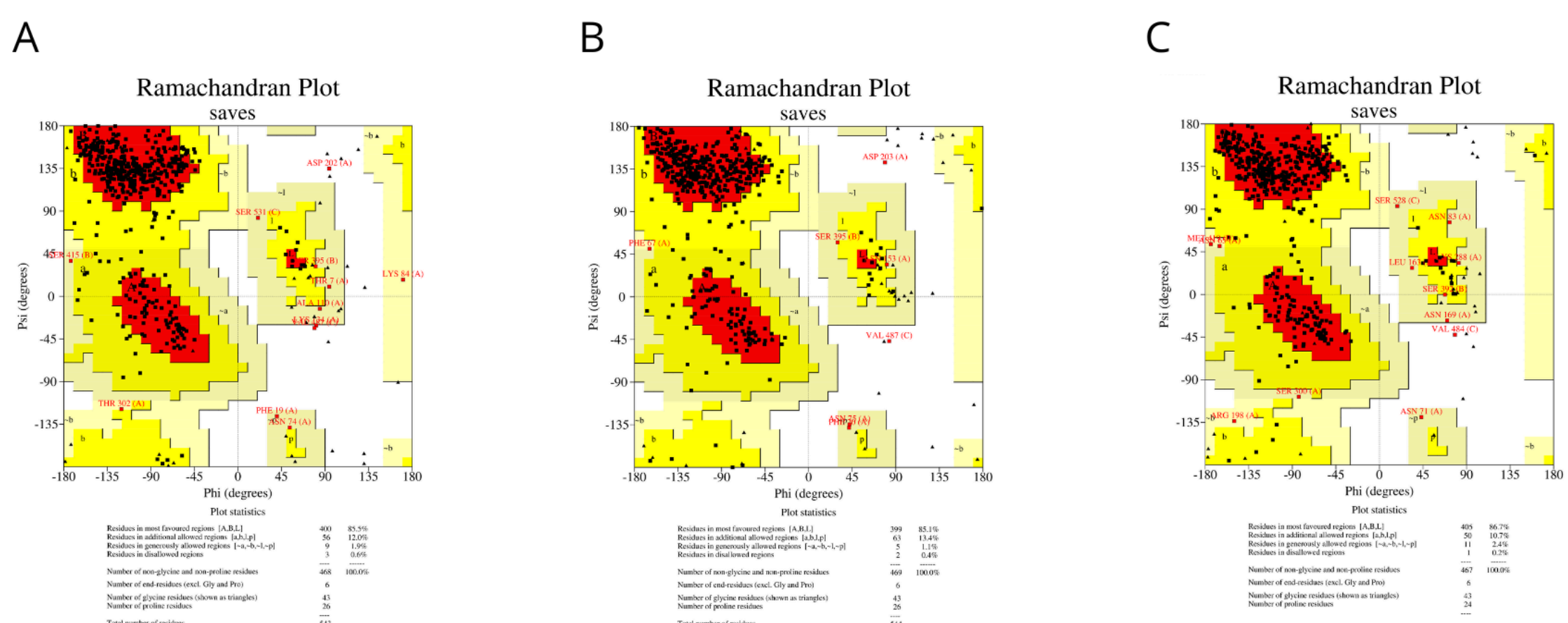

Supplementary Figure S2. Ramachandran plots (PROCHECK/SAVES) for the modeled NTD-*1-87* complexes: (A) WT, (B) Q23K, and (C) T19I/A27S/Δ24–26 (LPP). For each model, the plot statistics report the proportion of residues in the most-favoured, additional-allowed, generously-allowed, and

disallowed regions, supporting the stereochemical quality of the models used as starting structures for molecular dynamics.

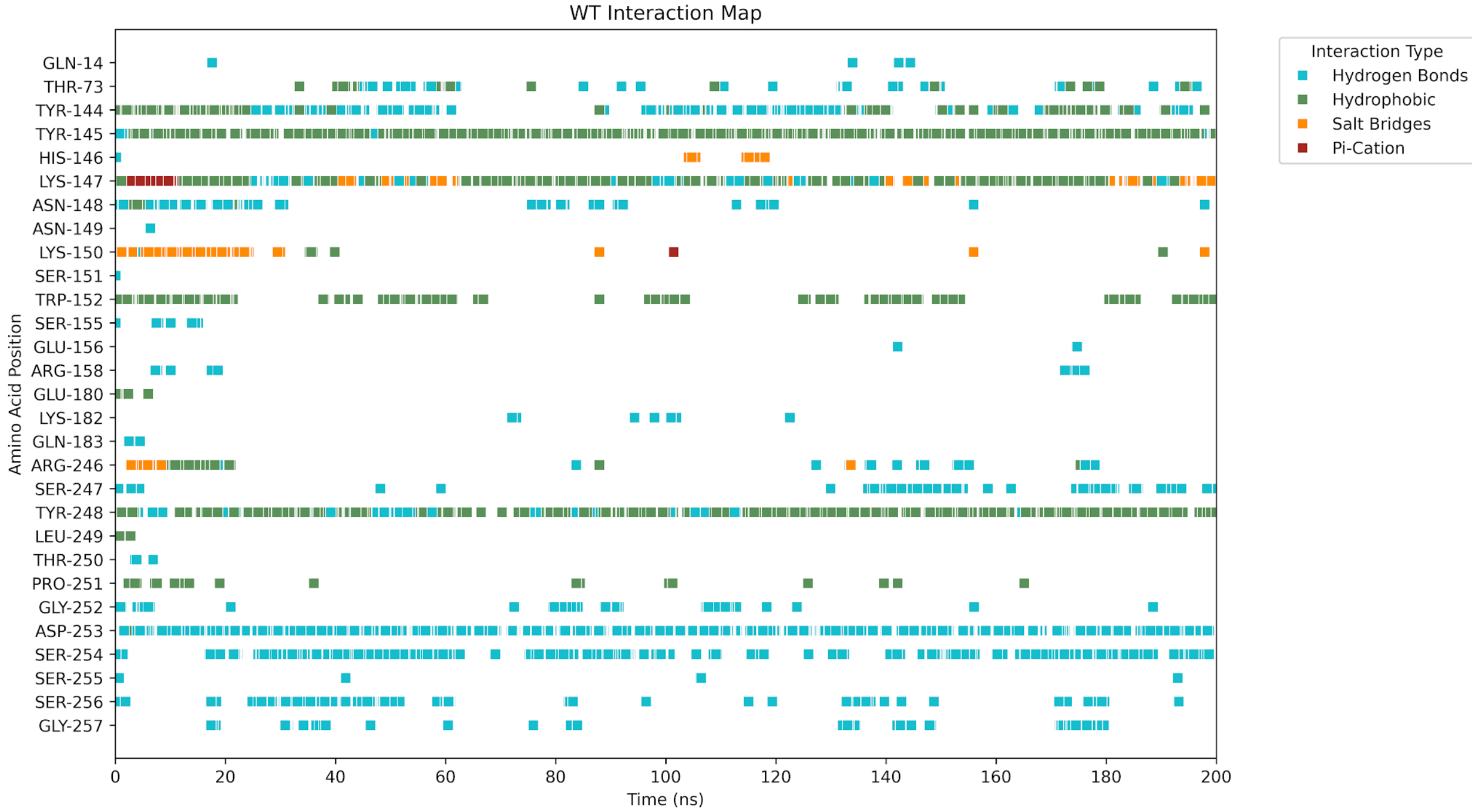

Supplementary Figure S3. Residue–time interaction map of the NTD-*1-87* interface in the WT system. Each row represents an NTD residue and colored marks indicate interaction events detected along the 200-ns trajectory, classified by interaction type (hydrogen bonds, hydrophobic contacts, salt bridges, π-cation, and π-stacking). This residue-centered temporal map complements the residue-pair persistence and hotspot summaries shown in Table III.

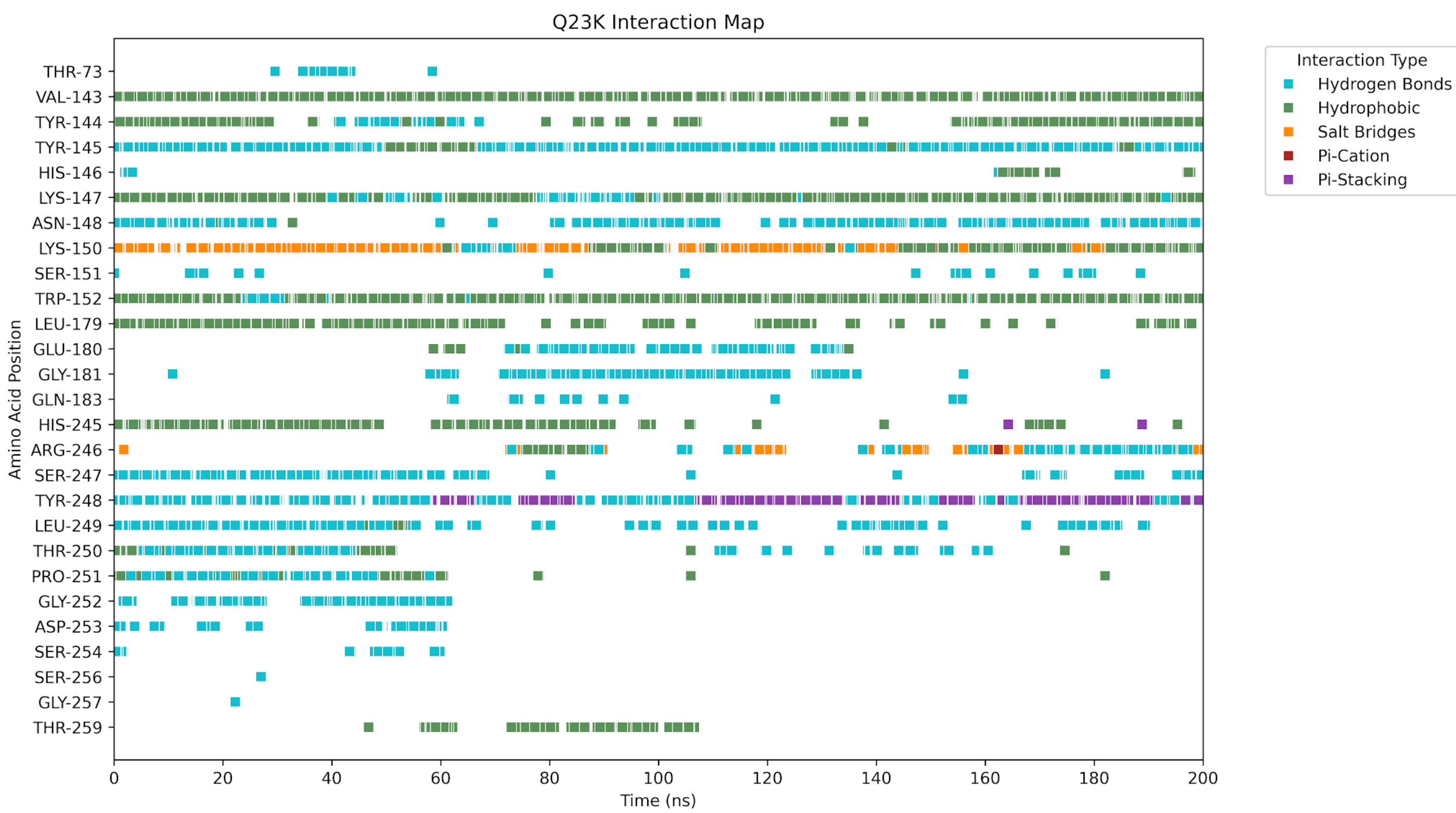

Supplementary Figure S4. Residue–time interaction map of the NTD-*1-87* interface in the Q23K system. Each row represents an NTD residue and colored marks indicate interaction events detected along the 200-ns trajectory, classified by interaction type (hydrogen bonds, hydrophobic contacts, salt bridges, π-cation, and π-stacking). This residue-centered temporal map complements the residue-pair persistence and hotspot summaries shown in Table III.

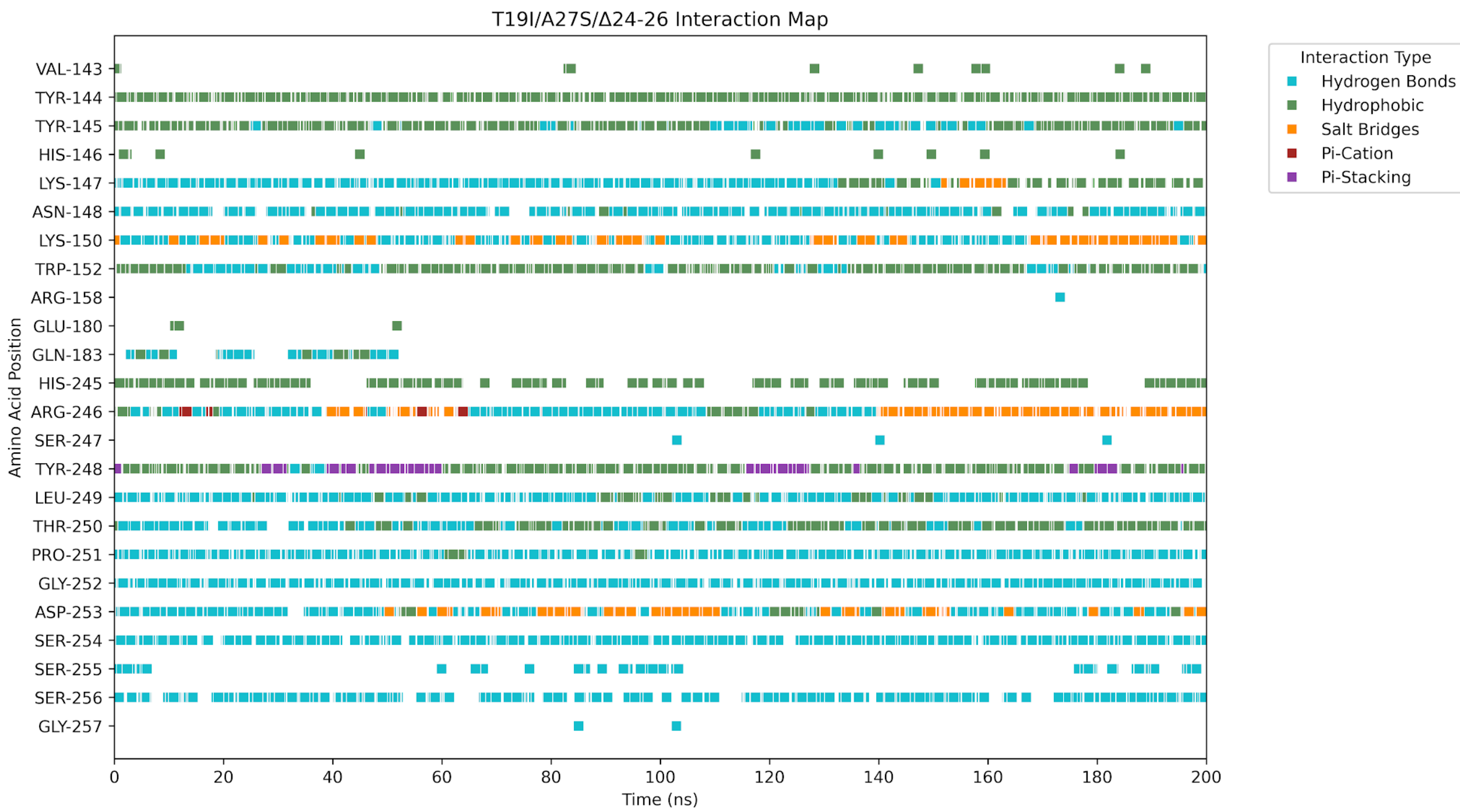

Supplementary Figure S5. Residue–time interaction map of the NTD-*1-87* interface in the T19I/A27S/Δ24–26 (LPP) system. Each row represents an NTD residue and colored marks indicate interaction events detected along the 200-ns trajectory, classified by interaction type (hydrogen bonds, hydrophobic contacts, salt bridges, π-cation, and π-stacking). This residue-centered temporal map complements the residue-pair persistence and hotspot summaries shown in Table III.

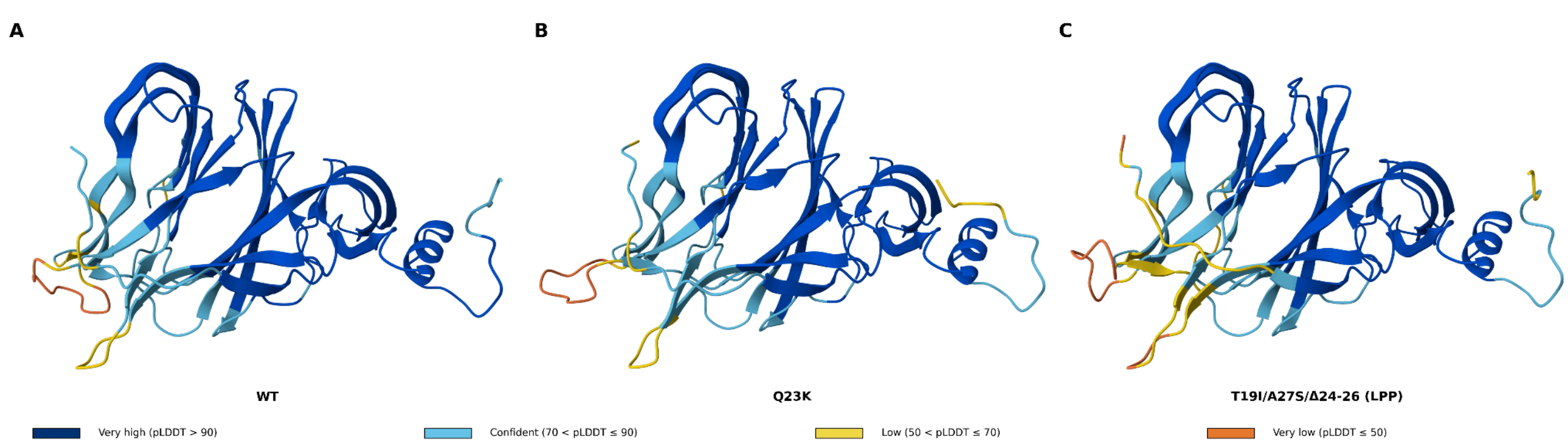

Supplementary Figure S6. AlphaFold3 per-residue confidence (pLDDT) of the modeled NTD structures for (A) WT, (B) Q23K, and (C) T19I/A27S/Δ24-26 (LPP), colored on a blue (very high) to orange (very low) scale. Overall pTM scores were 0.87 (WT), 0.85 (Q23K), and 0.84 (LPP).

Lower-confidence regions (yellow/orange) concentrate in loop segments, consistent with the supersite loops N3 and N5 being the least-determined regions of the model

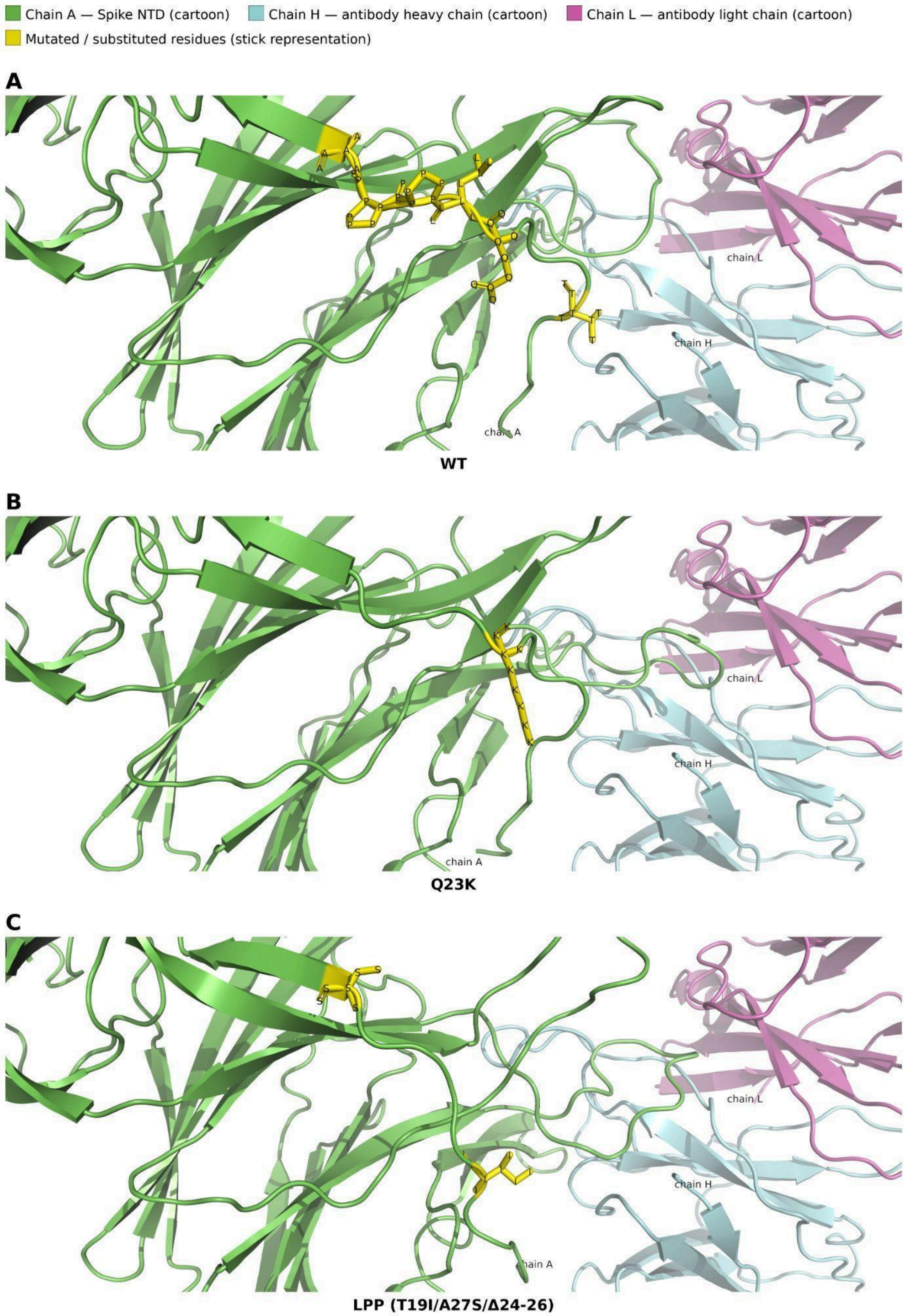

Supplementary Figure S7. Local structural context of NTD mutations at the *1-87* interface. (A) WT, (B) Q23K, and (C) LPP (T19I/A27S/Δ24-26) NTD-*1-87* complexes. Mutated residues are highlighted. Structural differences are confined to local loop rearrangements at the interface and are not apparent at the scale of the full complex. Quantitative changes in intermolecular contacts are reported in Figure 5 and Table III.
